# Insomnia, anxiety and related disorders: a systematic review on clinical and therapeutic perspective with potential mechanisms underlying their complex link

**DOI:** 10.1016/j.nsa.2024.103936

**Published:** 2024-01-07

**Authors:** Laura Palagini, Mario Miniati, Valerio Caruso, Gaspare Alfi, Pierre Alexis Geoffroy, Katharina Domschke, Dieter Riemann, Angelo Gemignani, Stefano Pini

**Affiliations:** a Department of Neuroscience, Psychiatric Section, Azienda Ospedaliera Universitaria Pisana (AUOP), Pisa, Italy; b Department of Surgical, Medical and Molecular Pathology, Critical and Care Medicine, University of Pisa, Pisa, Italy; c Département de Psychiatrie et d'addictologie, AP-HP, GHU Paris Nord, DMU Neurosciences, Hôpital Bichat - Claude Bernard, Paris, France; d Centre ChronoS, GHU Paris - Psychiatry & Neurosciences, Paris, France; e Université de Paris, NeuroDiderot, Inserm, Paris, France; f CNRS UPR 3212, Institute for Cellular and Integrative Neurosciences, Strasbourg, France; g Department of Clinical Psychology and Psychophysiology/ Sleep Medicine, Center for Mental Disorders, University of Freiburg, Freiburg, Germany

**Keywords:** Insomnia, GAD, PTSD, PD agoraphobia, Social anxiety, Separation anxiety, OCD

## Abstract

Anxiety and anxiety-related disorders are the most common mental disturbances, with dysregulation in emotions and cognition as central features. Since the function of sleep in regulating emotions, cognition stress response and inflammation is quintessential, sleep disturbances may be ideal modifiable factors in anxiety and related disorders. Accordingly, the aim of the review was to systematically review the association between insomnia symptoms and anxiety and related disorders. A systematic search has been conducted, and 93 papers have been selected for insomnia and General Anxiety Disorder, Panic Disorders, Social Anxiety Disorder, Separation anxiety, Obsessive Compulsive Disorder and Post Traumatic Stress Disorder, according to PRISMA. This review represents a comprehensive overview of clinical and therapeutic approaches to insomnia in the framework of anxiety and related disorders with a discussion of potential mechanisms underlying their complex link.

## 1 Introduction

Anxiety and anxiety-related disorders are the most common mental disturbances worldwide, with a 12-month prevalence of 14% among persons aged 14 to 65 (Ströhle et al., 2018). Anxiety disorders are the ninth leading cause of disability worldwide, according to the World Health Organization (WHO), due to their high incidence, chronicity, and comorbidity (Ströhle et al., 2018; Penninx et al., 2021). High prevalence, chronicity, and comorbidity led the World Health Organization (WHO) to rank anxiety disorders as the ninth most health-related cause of disability (Ströhle et al., 2018; Penninx et al., 2021). Accordingly, to indentifying modifiable markers of morbidity in order to prevent or improve trajectories of anxiety and related disorders is a priority.

Recent research has revealed the function of sleep in regulating emotions to be quintessential as well in regulating stress response and inflammation (Baglioni et al., 2010; Lo Martire et al., 2020; Akkaoui et al., 2023) with a good night of sleep being crucial for mental health. In fact, while sleep is essential for brain homeostasis and brain plasticity (Maier and Nissen, 2017) sleep disturbances, especially insomnia, may favor a state of allostatic overload impairing brain plasticity, emotional, immune and endocrine pathways and may play an anxiogenic role for the brain (Lo Martire et al., 2020) (Riemann et al., 2015; Hertenstein et al., 2019; Van Someren, 2021; Chellappa and Aeschbach, 2022; Palagini et al., 2022). Recent research suggests a bidirectional relationship, whereby symptoms of psychiatric disorders, including anxiety-related disorders, lead to disturbed sleep, and disturbed sleep, in particular insomnia, may represent a risk factor for the de novo onset of anxiety disorders (Hertenstein et al., 2019) (Chellappa and Aeschbach, 2022). In a recent meta-analysis, insomnia was the major predictor for the onset of anxiety-related disorder, with an odds ratio of 3.2 (Hertenstein et al., 2019). This may be confirmed by a recent two-sample Mendelian randomization study where genetically predicted insomnia statistically predicted anxiety and anxiety-related disorders (OR = 1.36, 95% CI = 1.23–1.51, P < 0.001) (Zhou et al., 2022).

There is robust evidence that, among sleep disturbances, insomnia frequently co-occurs with a wide range of psychiatric disorders including anxiety disorders and anxiety-related disorders (Chellappa and Aeschbach, 2022; Palagini et al., 2022). Insomnia symptoms are clinically significant features in almost 70–80 % of individuals experiencing anxiety, and it is a defining criterion of anxiety/anxiety-related disorders, including Generalized Anxiety Disorder (GAD) and Post traumatic stress disorder (PTSD) [14-10]. In this framework, insomnia can be related to illness severity and low responsiveness to treatment (Chellappa and Aeschbach, 2022; Palagini et al., 2022). On the other hand, targeting insomnia may be effective in reducing anxiety symptoms (Mirchandaney et al., 2022).

Based on this different evidence, this work aimed to focus on the clinical, neurobiological and therapeutic perspective of insomnia in the context of anxiety and anxiety-related disorders. To this aim, we performed a systematic search on the data about the clinical association between insomnia and anxiety/anxiety-related disorders according to DSM-5-TR classification (American Psychiatric Association, 2022) and included therapeutical implications with the discussion of potential mechanisms involved.

## 2 Methods

We conducted a systematic literature search on PubMed, PsycINFO and Embase electronic databases for English literature published until May 2023 according to PRISMA Guidelines (Page et al., 2021). The inclusion criteria were original studies exploring the association between insomnia and anxiety or anxiety-related disorders among patients under and over 18 years, with a longitudinal design; prospective or retrospective, observational (analytical or descriptive), experimental or quasi-experimental, controlled or non-controlled studies; articles accepted for publication in a peer-reviewed journal, written in English**.**

### 2.1 Exclusion criteria

(1) Absence of standardized measures of insomnia symptoms or disorder (questionnaires or DSM criteria); (2). Evaluation of other sleep disorders including poor sleep quality, sleep deprivation or sleep loss 3) papers including reviews and 4) and paper published not in English.

### 2.2 Information source and Search procedures

A search was conducted in May 2023, starting from 1990. PubMed database was searched, and the following queries in the *‘PubMed Advanced Search Builder’* were applied: **Search #1 (insomnia) AND (General Anxiety Disorders))** retrieved 304 papers, **Search # 2 ((insomnia) AND (Panic Disorder))** retrieved 22 papers, **Search:#3 ((insomnia) AND (agoraphobia))** retrieved 44 papers, **Search:#4 ((insomnia) AND (Social Anxiety Disorder)** retrieved 32 papers, **Search:#5 ((insomnia) AND (Specific Phobia))** retrieved 36 papers**, Search:#6 ((insomnia) AND (Separation Anxiety Disorder))** retrieved 40 papers, **Search #7 (insomnia) AND (Obsessive-compulsive disorder (OCD))** retrieved 38 papers **Search:#8((insomnia) AND (Post-traumatic stress disorder (PTSD))** 350 papers**, Search#9** (insomnia) AND (Selective Mutism) **28 papers.**

**Before to examine literature data on insomnia and anxiety and related disorders, we discussed current models of insomnia in a narrative way.**

## 3 Results

The literature search identified 24 records for insomnia comorbid with GAD, 14 for insomnia and PD with and without agoraphobia, 9 for Social Anxiety Disorder (SAD), 3 for Separation Anxiety (SA), 12 for Obsessive compulsive disorders (OCD), 43 for Post Traumatic Stress Disorders (PTSD). No studies interested insomnia and selective mutism and insomnia and specific phobia.

**Fig. 1:**
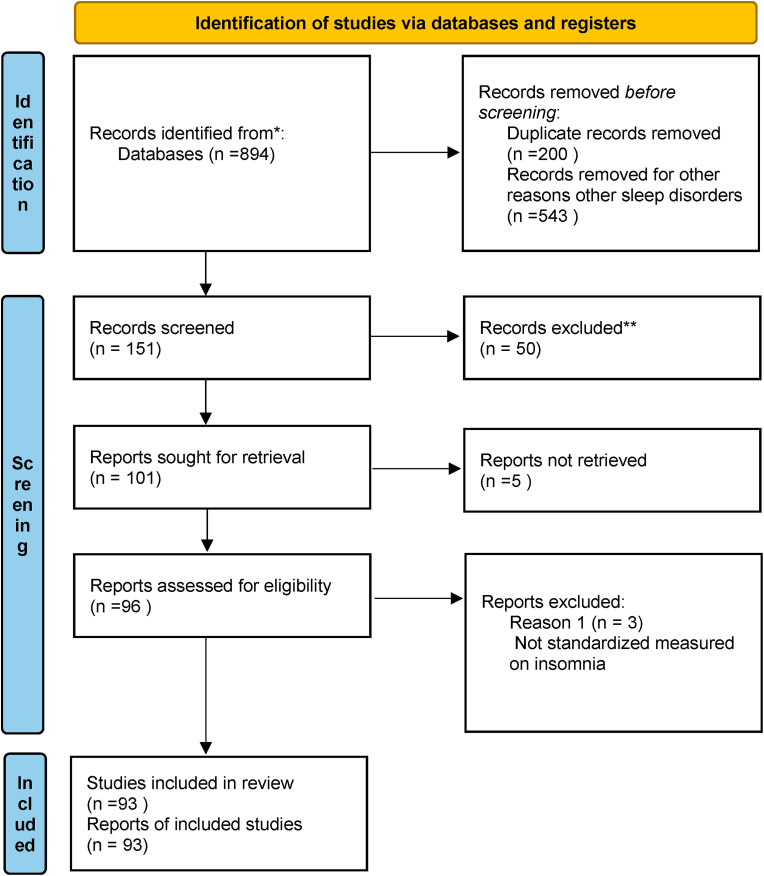
Prisma.

After removing duplicates, or papers not focused on the topic, a total of 93 paper were selected according to inclusion/exclusion criteria. Fig. 1 illustrates the selection of the studies in a PRISMA flow diagram (Page et al., 2021).

### 3.1 Current models of insomnia

Insomnia symptoms may be acute, transient and chronic. Chronic insomnia, also currently referred to as “insomnia disorder”, now has similar diagnostic criteria in the American Psychiatric Association's Diagnostic and Statistical Manual of Mental Disorders, Fifth Edition (DSM-5), and confirmed in the recent text revised version DSM-TR (American Psychiatric Association, 2022). Insomnia disorder is now considered a 24-h sleep-wake disorder characterized by nocturnal and diurnal symptoms. Approximately 6–10% of the European population with insomnia symptoms meets the diagnostic criteria for insomnia disorder (Riemann et al., 2022), while short-term insomnia may affect 30–50% of the population (Palagini et al., 2023a). Despite the fact that insomnia may be a symptom or an independent disorder, it is more often seen as co-morbid with another disease. Many co-occurring conditions, including anxiety and anxiety-related disorders, may be triggered and negatively affected by insomnia (Hertenstein et al., 2019). Following classification criteria (American Psychiatric Association, 2022) insomnia subjects suffer from both nocturnal and daytime symptoms such as fatigue, sleepiness, mood disturbance, subjective symptoms of distress, or impairment in psychosocial functioning. (American Psychiatric Association, 2022) (Riemann et al., 2022; Palagini et al., 2023a).

Current evidence for the pathophysiology of insomnia converges on the hypothesis that it is a complex polygenic stress-related disorder; a synergy of genetic and environmental factors within an epigenetic framework can be hypothesized for insomnia (Palagini et al., 2022) (Riemann et al., 2022; Palagini et al., 2023a). The hyperarousal, such as the hyperactivation of the arousal promoting neurotransmission consistent with the central and peripheral hyperactivation of stress and inflammatory systems has been shown in insomnia (Palagini et al., 2023a). Recent neuroimaging investigations have demonstrated that those with persistent insomnia have impaired amygdala functioning. Patients with insomnia have been shown to exhibit amygdala hyperactivity in reaction to cues associated with sleep (Riemann et al., 2015). Functional connectivity between the amygdala and premotor neurons is correlated with insomnia severity, and patients with insomnia have an abnormal amygdala-based intrinsic emotional network. Concerning structural neuroimaging, previous studies have shown a decreased brain volume in the medial frontal and middle temporal gyrus, middle cingulate cortex, and hippocampus (Riemann et al., 2015). Atrophy has been reported in subcortical structures, including the hippocampus, some areas of amygdala, basal ganglia, and thalamus in patients with chronic insomnia. Insomnia is therefore considered a stressor, which impairs neuroplasticity, leading to a state of allostatic overload with anxiogenic effect (Van Someren, 2021) (Riemann et al., 2022). These may result in disturbances with emotion regulation and decision-making processes, leading to emotional reactivity, impulsivity aggressive behaviours, and risky decisions (Riemann et al., 2022). Insomnia is hypothesized with a deficit in GABAergic synapses and a corresponding increase in cortical hyperarousal, both of which have been linked to hyperactivity of the stress axis. Some researchers believe that GABAergic abnormalities contribute to insomnia by causing maladaptive changes in neuroplasticity (Plante et al., 2012; Palagini and Bianchini, 2022). While sleep facilitates neural plasticity thought to consolidate newly acquired memories, in insomnia these processes may be impaired. In particular, insomnia is associated with reduced slow wake sleep and diminished sleep-related consolidation of memories (for an overview see (Palagini and Bianchini, 2022)). As impaired neuroplasticity favours the accumulation of neurotoxic proteins, neuroinflammation, and stress system alterations, insomnia is considered a causal factor in neurodegenerative and neuropsychiatric disorders (Palagini et al., 2022). Recent evidence has found that plasma orexin levels are significantly higher in patients with chronic insomnia compared with good sleepers (Tang et al., 2017). These data in humans may be confirmed by data obtained in animal studies in insomnia-like phenotypes. Data such as this support the hypothesis that insomnia can be due to an inability of the brain to switch off wake-promoting systems such as the orexin system, as well as an inability to switch on sleep promoting circuits with an instability of the flip-flop switch system (Palagini et al., 2022) (Riemann et al., 2022). Accordingly, it has been hypothesized that the overactivation of orexin system in insomnia may favor instability of the flip-flop switch system which may fuel hyperarousal (Palagini et al., 2023b).

Another hypothesis for insomnia recognizes the role of melatonin. The production of melatonin generally decreases with age, and it is related to an increase in insomnia prevalence in the elderly. The “melatonin-replacement” theory proposes that decreasing melatonin synthesis with age contributes to an increase in the occurrence of insomnia. Indeed, in young people with insomnia, a low melatonin level has been observed as well (Riemann et al., 2022). Accordingly, dysfunctions in melatonin production and rhythms have been hypothesized to contribute to insomnia chronicity. Cognitive and behavioral models of insomnia also hypothesized hyperarousal to interact with unhelpful cognitive beliefs about sleep and to favor negative behaviours which contribute to the perpetuation of insomnia (Riemann et al., 2022). In summary, cognitive, behavioral and neurobiological hypotheses converge on the idea that insomnia is a disorder of hyperarousal that may explain night-time and daytime symptoms (Riemann et al., 2022). According with international guidelines, primary treatment goals for insomnia should be to improve insomnia-related nighttime and daytime symptoms. While for acute-transient forms of insomnia pharmacological treatment remain the first line approach (Sateia et al., 2017; Wilson et al., 2019; Riemann et al., 2023a), for chronic insomnia the Cognitive Behavioral Therapy-for Insomnia (CBT-I) is the internationally considered first line treatment (Sateia et al., 2017; Wilson et al., 2019; Riemann et al., 2023a; Palagini et al., 2023). Available guidelines and reviews for insomnia treatment include sleep-promoting gamma-aminobutyric acid (GABA)A receptor agonists, such as benzodiazepines and Z-drugs, including zolpidem, zaleplon zopiclone, and eszopiclone. Among sleep-promoting compounds melatonin receptor agonists M1 and M2 such as melatonin 2 mg-prolonged release (PR) and ramelteon, represent more recent important therapeutic options. Antagonists of the arousal-promoting system such as antidepressants, neuroleptics or antihistaminergic compounds with histaminic or 5HT2 antagonism have been used for decades in the treatment of insomnia (Sateia et al., 2017; Riemann et al., 2023a). Still, they are not indicated for insomnia treatment. Among them, only doxepine is indicated for insomnia treatment (Sateia et al., 2017). New options as antagonists of the arousal promoting systems approved for chronic insomnia treatment include Dual Orexin Receptors Antagonists (DORAs) daridorexant, suvorexant and lemborexant. Daridorexant is the first DORA approved in Europe for chronic insomnia treatment (Sateia et al., 2017).

### 3.2 Insomnia, anxiety and anxiety-related disorders: a systematic review of clinical and therapeutic approach

#### 3.2.1 Insomnia and generalized anxiety disorder

Common anxiety disorders include generalized anxiety disorder (GAD). Anxiety disorders have been estimated to have a prevalence of 10%–25% during the lifetime of an individual GAD DSM-5 criteria include having excessive anxiety and worry (apprehensive expectation), difficulty controlling the worry, and association of the anxiety and worry with three (or more) of physical and cognitive symptoms of restlessness, feeling keyed up or on edge, being easily fatigued, difficulty concentrating or mind going blank, irritability, muscle tension, and sleep disturbance (American Psychiatric Association, 2022). Insomnia is one of the key diagnostic criteria for GAD starting from DSM-III. Papers selected based on inclusion/exclusion criteria for insomnia comorbid were 22 (Table 1) (Palagini et al., 2021; Ferre Navarrete et al., 2017; Breslau et al., 1996; Ohayon, 1997; Alfano et al., 2010; Soehner and Harvey, 2012; Steinsbekk et al., 2013a; Ivan et al., 2014; Alvaro et al., 2014; Pace-Schott et al., 2015; Li et al., 2019; Saletu et al., 1994, 1997; Bélanger et al., 2004; Pollack et al., 2008; Fava et al., 2009; Montgomery et al., 2009; Krystal et al., 2012; Mason and Harvey, 2014; Belleville et al., 2016; Cousineau et al., 2016; Jansson-Fröjmark and Jacobson, 2021; Huang et al., 2018; Kaczkurkin et al., 2021).

**Table 1:** Studies on insomnia and Generalized Anxiety Disorder (GAD).

Authors	Sample and Study design	Assessment anxiety and insomnia	Main findings
Generalized Anxiety Disorder (GAD)			
Breslau et al., 1996	n = 1007	DSM-III-R	The lifetime prevalence of insomnia was 16.6%. OR for GAD 7. (2.8–17.2) combined with hypersomnia 4.8 (1.5–15.2)
	18–65 years Health Maintenance Organization members	Sleep complains Lifetime	
Ohayon, 1997	n = 5622	DSM-IV	A total of 18.6% of the sample complained of insomnia. Insomnia related to another mental disorder accounted for 2.9% of the sample, and this was primarily a major depression (1.3%) or an anxiety disorder (0.7%).
	15–96 years general population	epidemiological surveys in mental health	
	Cross-sectional	Eval	
Brenes et al., 2009, Palagini et al. (2021)	n = 110 adults	SCID-IV	4.6% of individuals with no diagnosis, 16% of worried well, 51% of GAD patients, and 68% of patients with GAD and depression reported having insomnia symptoms. The most common sleep complaint in GAD and GAD + depression (>80%) was difficulty remaining asleep
	31 with GAD, 25 with GAD and depression, 33 worried well, and 21 with no psychiatric diagnosis.	Insomnia Severity Index (ISI)	
	Cross-sectional		
Alfano et al., 2010	n = 52 anxious children and adolescents aged 7–14 years	Anxiety Disorders Interview Schedule for DSM-IV:Child and Parent Versions (ADIS-C/P)	A significantly greater proportion of youth with GAD (87%) reported difficulty sleeping compared to youth with all other primary anxiety diagnoses (60% SAD, 27% SP and 54% OCD) [χ^2^(3) = 9.45, p = 0.02].
	Cross-sectional	Children's Sleep Habits Questionnaire (CSHQ)	
Soehner et al., 2012 Soehner and Harvey (2012)	N = 5692 respondents with no mood or anxiety disorder (n = 3711), mood disorders only (n = 327), anxiety disorders only (n = 1137), and coexisting mood and anxiety disorders (n = 517) retrospective	DSM-IV criteria in the past year	Rates of insomnia were 15% in patients with GAD
Steinsbekk et al., 2013 Steinsbekk et al. (2013a)	N = 1250 children born in 2003 or 2004 in Trondheim, Norway, who attended regular community health checkups for 4 year olds longitudinal	DSM-IV criteria	Primary insomnia,16.6% was specifically related to symptoms of depression, generalized anxiety disorder, separation anxiety, and specific phobia
[Bibr bib58][Bibr bib58]	N = 223 patients with GAD age 60 and older	DSM-IV	Alcohol usage seems to be higher among older persons with GAD and insomnia. In older persons with GAD, the relationship between anxiety/worry and sleeplessness was reduced to a lesser extent when alcohol was consumed in moderation
		Insomnia Severity Index (ISI)	
		General Anxiety Disorder-7 (GAD-7)	
[Bibr bib5][Bibr bib5]	N = 318	DSM-IV	After confounder variables controlled insomnia was predicted by depression and GAD
	South Australian high school students from grades cross-sectional study	Insomnia Severity Index (ISI)	
Pace-Schott et al., 2017 Pace-Schott et al. (2015)	N = 13 individuals with primary insomnia	DSM-IV-TR wrist actigraphy and sleep diaries	With underlying Primary Insomnia, less top-down regulation of the amygdala may increase the chance of developing GAD
	N = 11 individuals with GAD	Resting state functional connectivity analysis	
		3-T Siemens Magnetom TrioTim Syngo scanner	
Ferre-Navarrete et al., 2017 Ferre Navarrete et al. (2017)	N = 840	General Anxiety Disorder-7 (GAD-7)	Insomnia was prevalent in GAD at 85.3%. Females were more likely than men to have insomnia. Increased severity of insomnia was linked to increased severity of GAD. The most prevalent forms of sleep disorders associated with insomnia were difficulty falling asleep (36.6%) and sleep maintenance (33.0%). In instance, 55.5% of GAD patients reported that their insomnia significantly hampered their daily functioning.
	patients with GAD and insomnia	Insomnia Severity Index (ISI)	
Li et al., 2019 Li et al. (2019)	N = 7 patients with GAD with insomnia(GAD/IS), N = 15 GAD	DSM-5	Patients with GAD/IS exhibited substantially increased network segregation and hyperaroused posterior cingulate cortex, as well as significant associations with anxiety and insomnia severity.
	N = 24 healthy controls cross-sectional	Functional magnetic resonance imaging (fMRI)	
		Hamilton Anxiety Rating Scale (HAMA)	
		Insomnia Severity Index (ISI)	
**GAD and insomnia treatment**			
Saletu et al., 1994 Saletu et al. (1994)	N°45 patients with insomnia and GAD comparative study on the effects of quazepam and triazolam	ICD-9	Both quazepam and triazolam were effective in reducing anxiety, with quazepam being marginally more effective than triazolam. Anxiety remained significantly decreased for two weeks following placebo administration.
		Hamilton Anxiety Rating Scale (HAMA) sleep polysomnography	
[Bibr bib127][Bibr bib127]	N°44 patients with insomnia and GAD	ICD-9	Anxiety improved in observer ratings (HAMA) under both drugs, in self-rating under the combination drug only
	Comparative study on the effect of lorazepam plus diphenhydramin versus lorazepam alone	Hamilton Anxiety Rating Scale (HAMA) sleep polysomnography	
[Bibr bib15][Bibr bib15]	N°44 GAD patients with insomnia	Structured clinical interview and the Anxiety Disorder Interview Schedule-IV (ADIS-IV).	47.7% reported difficulties initiating sleep, 63.6% reported difficulties maintaining sleep, and 56.8% complained of waking too early in the morning. CBT package for GAD had a significant impact on sleep quality even if sleep disturbances were not specifically addressed during treatment but insomnia symptoms persisted after CBT for GAD in some patients
	Underwent GAD CBT longtudinal	Insomnia Severity Index (ISI),	
[Bibr bib112][Bibr bib112]	N = 595 adults aged 18–64 years with GAD and insomnia	DSM-IV-TR criteria for GAD and insomnia.	Treatment with eszopiclone and escitalopram resulted in significantly better sleep and daytime functioning (P 0.05), with no evidence of tolerance. Patients taking eszopiclone and escitalopram had greater improvements in total Hamilton Anxiety Scale (HAM-A) scores at each week (P < 0.05) and at weeks 4 through 10 with the insomnia item removed. Clinical Global Impressions (CGI) was improved with eszopiclone and escitalopram at every point (P < 0.02).After eszopiclone discontinuation, there was no evidence of rebound insomnia, and while treatment differences in anxiety measures were maintained, differences in sleep outcomes were not
	Double-blind, randomized, placebo-controlled, parallel-group, add-on therapy 10-week study.	Hamilton Anxiety Rating Scale (HAMA)	
	Patients received 10 mg of escitalopram for 10 weeks and were randomized to also receive either 3 mg of eszopiclone (n = 294) or placebo (n = 301) nightly for 8 weeks. For the last 2 weeks, eszopiclone was replaced with a single-blind placebo longitudinal		
[Bibr bib38][Bibr bib38]	N° 383 patients with insomnia and comorbid GAD. received open-label escitalopram 10 mg/d and were randomized to either adjunct zolpidem extended-release 12.5 mg or placebo longitudinal	Hamilton Anxiety Rating Scale (HAMA)	In patients with concurrent insomnia and GAD, concurrent zolpidem extended-release/escitalopram significantly improved insomnia and sleep-related next-day symptoms, but not anxiety symptoms, at week 8
		Sleep Impact Scale	
Montgomery et al., 2009 (Montgomery et al., 2009)	N = 2004 outpatients	DSM-IV	Pregabalin 300–450 mg was well tolerated, and improved overall anxiety symptoms, and insomnia in patients with GAD
	Six double-blind, placebo-controlled, 4- to 6-week trials of three fixed-dose pregabalin (150, 300–450, 600 mg/day), alprazolam lorazepam longitudinal	Hamilton Rating Scale for Anxiety (HAM-A)	
Krystal et al., 2012 (Krystal et al., 2012)	GAD	DSM-IV-TR Insomnia Severity Index (ISI)	At week 4, effect sizes on Insomnia Severity Index tended to be lowest for patients with comorbid GAD and MDD. The effect sizes for daytime functioning were small for all insomnia patient groups
	Primary Insomnia		
	Major Depressive Disorder (MDD)		
	5 large, multicenter, randomized, double-blind, placebo-controlled studies of adult outpatients of at least 1 month duration published between 2006 and 2009 eszopiclone 3 mg vs placebo ongitudinal		
Mason et al., 2014 (Mason and Harvey, 2014)	N = 266 patients with anxiety related disorders	General Anxiety Disorder-7 (GAD-7)	Patients with GAD (12% of the sample) experienced insomnia symptoms in the 38% of the cases. Those with insomnia reported more severe anxiety and depression symptoms than those without insomnia. CBT focusing on anxiety and/or depression was associated with reductions in insomnia, anxiety, depression, and disability symptoms, but had no effect on sleep duration.
	Including GAD	Insomnia Severity Index (ISI)	
	Underwent CBT for anxiety disorders		
Belleville et al., 2016 Belleville et al. (2016)	N = 10 women (mean age = 45) with chronic insomnia and GAD were randomly assigned to CBT for GAD followed by CBT for insomnia, or to CBT for insomnia followed by CBT for GAD. Single-case methodology	Insomnia Severity Index	In the presence of both GAD and insomnia, initiating GAD treatment first produced superior anxiety and sleep outcomes. The inclusion of insomnia-specific treatment resulted in further enhancements to both anxiety and sleep quality.
Cousineau et al., 2016 (Cousineau et al., 2016)	N = 86 participants a quasi-experimental design patients with agoraphobia PDA, GAD and insomnia received conventional CBT for their primary disorder or combined CBT for both disorders	Insomnia Severity Index.	CBTs had a significant impact on reducing insomnia symptoms (η = 0.58) in patients with GAD. However, among people with insomnia at pretest (67%), 33% still had an insomnia diagnosis, and the majority (63%) had clinically significant residual insomnia following treatment. CBTs had a positive effect on the reduction of insomnia, but a significant proportion of participants still had insomnia problems following treatment. Clinicians should address insomnia during CBT for GAD.
Jansson-Fröjmark et al., 2021 (Jansson-Fröjmark and Jacobson, 2021)	N = 23 patients with insomnia disorder co-morbid with GAD efficacy of CBT-Insomnia	Mini-International Neuropsychiatric Interview (MINI)	For insomnia symptoms, moderate to large effect sizes for CBT-I were observed. Regarding the severity of insomnia, approximately 61% of patients responded to CBT-I, and between 26% and 48% remitted. Effect sizes ranging from moderate to large were also observed for GAD symptoms, depression, functional impairment, and quality of life.
		Insomnia Severity Index	
		Generalized Anxiety Disorder Screener (GAD-7)	
Huang et al., 2018 (Huang et al., 2018)	N°36 patients with GAD and Insomnia were randomized to either sham or active Repetitive transcranial magnetic stimulation	DSM-IV	Results suggested that 1 Hz low frequency rTMS administered over the parietal cortex is effective for both anxiety and insomnia symptoms in patients with comorbid GAD and insomnia.
	rTMS group (n = 18 each group)	Hamilton Rating Scale for Anxiety (HAM-A)	
Kaczkurkin et al., 2021 (Kaczkurkin et al., 2021)	N = 326 patients seeking treatment at a clinic specializing in CBT for anxiety	DSM-5	After controlling for the overlap between anxiety symptom and depressive symptom classes, GAD was significantly associated with insomnia. Insomnia symptoms may persist following treatment for anxiety, suggesting that CBT for insomnia may be necessary during or after a course of CBT for anxiety.
		Insomnia Severity Index	
		Generalized Anxiety Disorder, 7-item scale (GAD-7)	

Available data show that insomnia is associated with increased odds of GAD (Palagini et al., 2021; Breslau et al., 1996; Steinsbekk et al., 2013a). Though scarcely investigated, insomnia has been reported from 15% to 85% of patients with GAD among adults and up to 87% in children and adolescents (Palagini et al., 2021; Ferre Navarrete et al., 2017; Breslau et al., 1996; Ohayon, 1997; Alfano et al., 2010; Soehner and Harvey, 2012; Steinsbekk et al., 2013a). Insomnia was related to GAD severity and higher risk of comorbidities, such as depressive symptoms and alcohol use (Ferre Navarrete et al., 2017; Breslau et al., 1996; Ohayon, 1997; Alfano et al., 2010; Soehner and Harvey, 2012; Steinsbekk et al., 2013a; Ivan et al., 2014) and with resistance to GAD treatments (Pollack et al., 2008; Mason and Harvey, 2014; Belleville et al., 2016; Cousineau et al., 2016; Jansson-Fröjmark and Jacobson, 2021; Kaczkurkin et al., 2021). Different therapeutic approaches have been proposed for insomnia treatment in GAD; in particular, CBT-Insomnia and eszopiclone, when combined with GAD therapy, have been indicated as effective in treating both insomnia and GAD symptomatology, while other therapeutical approaches have been proposed, such as gabapentin, pregabalin or rTMS but in few studies (Saletu et al., 1994, 1997; Bélanger et al., 2004; Pollack et al., 2008; Fava et al., 2009; Montgomery et al., 2009; Krystal et al., 2012; Mason and Harvey, 2014; Belleville et al., 2016; Cousineau et al., 2016; Jansson-Fröjmark and Jacobson, 2021; Huang et al., 2018; Kaczkurkin et al., 2021) (Table 1).

Neuroimaging studies on insomnia and GAD have shown that abnormal neuroplasticity and dysfunctional insomnia-networking may predispose to hyperarousal and to alterations in the top-down regulation of amygdale functioning may be contributing to GAD symptomatology (Pace-Schott et al., 2015; Li et al., 2019).

#### 3.2.2 Insomnia, panic disorder and agoraphobia

Panic Disorder (PD) is characterized by repeated, unexpected panic attacks and by persistent concern about subsequent attacks (American Psychiatric Association, 2022). Few studies have explored insomnia symptoms in panic disorder (PD) with and without agoraphobia. In particular, 14 studies only were selected according to inclusion/exclusion criteria (Table 2) (Breslau et al., 1996; Soehner and Harvey, 2012; Alvaro et al., 2014; Mason and Harvey, 2014; Cousineau et al., 2016; Kaczkurkin et al., 2021; Ağargün and Kara, 1998; Babson et al., 2008; Pattyn et al., 2015; Park et al., 2021; Hong et al., 2021; Saletu-Zyhlarz et al., 2000; Cervena et al., 2005).

**Table 2:** Studies on insomnia and Panic Disorder and Agoraphobia.

Panic Disorder (PD)			
Breslau et al., 1996 (Breslau et al., 1996)	n = 1007	DSM-III-R	The lifetime prevalence of insomnia was 16.6%. OR for PD 5.3 (2.0–13.6) combined with hypersomnia 8.5 (3.1–15.2)
	18–65 years Health Maintenance Organization members longitudinal	Sleep complains Lifetime	
Ağargün et al., 1998 Ağargün and Kara (1998)	N = 33 sleep panickers	DSM-III-R	Recurrent sleep panickers also had a higher percentage of insomnia and comorbid major depression than the others.
	N = 34 other panickers	Schedule for Affective Disorders and Schizophrenia (SADS)	
	Cross-sectional		
[Bibr bib12][Bibr bib12]	N = 5692	DSM-IV	In a nationally representative sample, nicotine dependence was a partial mediator of the associations between insomnia symptoms and both PD and PTSD. Overall, the results imply that nicotine dependence may be a potential mechanism underlying insomnia in PD and PTSD patients.
	(3311 females) adults from the National Comorbidity Survey-Replication	Insomnia Severity Index (ISI)	
	Cross-sectiona		
Soehner et al., 2012 (Soehner and Harvey, 2012)	N = 5692 respondents with no mood or anxiety disorder (n = 3711), mood disorders only (n = 327), anxiety disorders only (n = 1137), and coexisting mood and anxiety disorders (n = 517)	DSM-IV criteria in the past year	Rates of insomnia complains were 12% in patients with PD and 4.1 in patients with agoraphobia and PD.
	Cross-sectional		
Alvaro et al., 2014 (Alvaro et al., 2014)	N = 318 high school students from grades South Australian cross-sectional	DSM-IV	After confounder variables were controlled, insomnia predicted depression and PD eveningness predicted the models in which depression and PD predicted insomnia and vice versa.
		Insomnia severity idex	
Pattyn et al., 2015 (Pattyn et al., 2015)	N = 658 PD population sample from the Netherlands Study of Depression and Anxiety (NESDA)	DSM-IV	Insomnia was associated to the severity of cognitive-autonomic subtype
	Cross-sectional	Insomnia severity idex	
Page et al., 2021Park et al. (2021)	N = 1025,340 individuals KNHIS National Sample Cohort 2002–2013	ICD-10: F41.0 for PD and ICD-10: G47	Patients with sleep disorders had higher incidence of panic disorder. In particular, patients with insomnia had the strongest association with panic disorder (adjusted, OR, 1.386; 95% CI, 1.201–1.599; p < 0.05).
	N = 7436 patients with PD and N = 21,876 gender- and age-matched as controls.	For sleep disorders	
	Cross-sectional		
Hong et al., 2021 (Hong et al., 2021)	N = 110	DSM-5	Of the PD patients, 88 (80%) and 89 (80.9%) had comorbid depression (and insomnia, respectively. In a mediation model using insomnia as the mediating variable, the total effect of panic symptom severity on depression was significant (t = 7.23, P < 0.001). There were significant effects of panic symptoms on insomnia (t = 4.62, P < 0.001) and of insomnia on depression (t = 6.69, P < 0.001). The main effect of panic symptom severity on depression was also significant, after controlling for the effect of insomnia (t = 5.10, P < 0.001), suggesting partial mediation.
	Patients with	PD Severity Scale (PDSS)	
	PD	Insomnia Severity Index	
**Insomnia treatment and PD**			
Saletu et al., 2000 (Saletu-Zyhlarz et al., 2000)	N = 11 drug-free patients (4 females, 7 males) aged 30–55 years with nonorganic insomnia related to panic disorder compared with N = 11 age- and sex-matched normal controls aged 30–58 years alprazolam vs placebo longitudina	polysomnography	alprazolam normalize sleep and awakening quality in patients with insomnia and PD
Cervena et al., 2005 (Cervena et al., 2005)	N = 20 successive outpatients (14 females and 6 males) aged 19–50 with PD Agoraphobia was diagnosed in 11 cases. In 16 of the cases, the panic disorder was accompanied by chronic insomnia. Patients were under treatment, psychotherapy, or benzodiazepines or SSRI	DSM IV	A statistical comparison of data revealed that a reduction in anxiety following the successful treatment of panic disorder was not necessarily accompanied by an improvement in sleep parameters. The findings indicate that the conventional therapy administered to these patients is insufficient to alleviate the concurrent insomnia
	Cross-sectional	Beck Anxiety Inventory (BAI) polisomnoghraphy	
Mason et al., 2014 (Mason and Harvey, 2014)	N = 266 patients with anxiety related disorders	Panic Disorder Symptom Scale, Self Report form (PDSS-SR)	Patients with PD 10.62%, 27% experienced insomnia. Individuals with insomnia reported more severe symptoms of anxiety and depression than individuals without insomnia. CBT focused on anxiety and/or depression was associated with reductions in symptoms of insomnia, anxiety, depression, and disability but it was ineffective in improving sleep duration
	Including PD with and without aghoraphobia	Insomnia Severity Index	
	Underwent to CBT for anxiety disorders		
Cousineau et al., 2016 (Cousineau et al., 2016)	N = 86 a quasi-experimental design, participants with agoraphobia Panic Disorder (PDA) received conventional CBT for their primary disorder or combined CBT for both disorders longitudinal	Sleep and anxiety were measured via diagnostic interviews, daily diaries, and self-report questionnaires	CBTs had a significant impact on reducing insomnia symptoms (η = 0.58) in patients with PDA. However, among people with insomnia at pretest (67%), 33% still had an insomnia diagnosis, and the majority (63%) had clinically significant residual insomnia following treatment. CBTs had a positive effect on the reduction of insomnia, but a significant proportion of participants still had insomnia problems following treatment. Clinicians should address insomnia during CBT for PDA.
		Insomnia Severity Index	
Kaczkurkin et al., 2021 (Kaczkurkin et al., 2021)	N = 326 patients seeking treatment at a clinic specializing in CBT for anxiety	DSM-5	Insomnia symptoms may persist following treatment for anxiety, suggesting that CBT for insomnia may be necessary during or after a course of CBT for anxiety.
		Insomnia Severity Index	
**Agoraphobia**			
Soehner et al., 2012 (Soehner and Harvey, 2012)	N = 5692 NCS-R respondents with no mood or anxiety disorder (n = 3711), mood disorders only (n = 327), anxiety disorders only (n = 1137), and coexisting mood and anxiety disorders (n = 517)	DSM-IV criteria in the past year	Rates of insomnia complains were 5.2 in patients with agoraphobia without PD
	Cross-sectional		

Available data show that Insomnia symptoms are associated with increased odds of PD and agoraphobia (Breslau et al., 1996; Park et al., 2021) and their frequency may range from 12% to more than 80 % in patients with PD (Soehner and Harvey, 2012; Hong et al., 2021). There is also support for the link between insomnia and panic disorder severity (Babson et al., 2008; Pattyn et al., 2015; Park et al., 2021; Hong et al., 2021). In PD, insomnia symptoms may probably mediate the link with depression (Baglioni et al., 2010; Hong et al., 2021) and resistance to PD treatments (Mason and Harvey, 2014; Cousineau et al., 2016; Cervena et al., 2005). Although a specific therapeutic approach for insomnia in PD has been suggested, no studies have explored the topic.

Most sleep studies in PD included the study of poor sleep quality or sleep complains or sleep disturbances or sleep duration but they were not measured with specific questionnaires (Roth et al., 2006; Singareddy and Uhde, 2009; Todder and Baune, 2010; Batterham et al., 2012; Swinkels et al., 2013). Indeed, all of them found a link between disturbed sleep and PD severity.

No studies systematically explored the association between insomnia and nocturnal panic attacks.

In 1998, Agargun et al. (Ağargün and Kara, 1998) showed that nocturnal panic attackers had more insomnia than the other panic disorder patients (Table 1). In another study, sleep complaints were detected in PD. They showed that 77% of the PD patients with nocturnal panic attacks reported sleep complaints versus 53% of the PD patients without nocturnal panic (Overbeek et al., 2005). Since fear of sleep and loss of vigilance may be a feature of nocturnal panickers from daytime panickers (Smith and Capron, 2021) insomnia may be more frequent in patients with nocturnal attacks, since they may avoid going to sleep. Indeed, more studies are needed in this field.

Another topic that is poorly studied regards the association between insomnia and agoraphobia with and without PD. Two studies interested agoraphobia. Results showed that insomnia symptoms might range from 4.1% in patients with agoraphobia and PD to 5.2 % in patients with agoraphobia without PD (Soehner and Harvey, 2012). In another study, CBT for anxiety showed a positive effect on the reduction of insomnia in patients with agoraphobia and PD, but a significant proportion of participants still had insomnia problems following treatment. Authors suggested that clinicians should address insomnia during CBT for Panic Disorder Agoraphobia (Kaczkurkin et al., 2021).

#### 3.2.3 Social anxiety disorder

DSM-5 criteria for social anxiety disorder (SAD) include: persistent, intense fear or anxiety about specific social situations because you believe you may be judged negatively, embarrassed or humiliated. Avoidance of anxiety-producing social situations or enduring them with intense fear or anxiety (American Psychiatric Association, 2022). Few studies have examined insomnia symptoms in SAD. In particular, 9 studies only were selected according to inclusion/exclusion criteria (Table 3) (Soehner and Harvey, 2012; Alvaro et al., 2014, 2017; Mason and Harvey, 2014; Buckner et al., 2008; Raffray et al., 2011; Steinsbekk and Wichstrøm, 2015; Blumenthal et al., 2019; Tang, 2010). Insomnia symptoms emerged as a risk factor for SAD and vice versa (Alvaro et al., 2014, 2017; Steinsbekk and Wichstrøm, 2015).

**Table 3:** Studies on insomnia and Social Anxiety Disorder (SAD).

Social Anxiety Disorder (SAD)			
Buckner et al., 2008 (Buckner et al., 2008)	n = 176 College students cross-sectional	Insomnia Severity Index	The relationship between social anxiety and insomnia is mediated by a positive correlation between social anxiety and insomnia with depressive symptoms.
Raffray et al., 2011 (Raffray et al., 2011)	N = 191 patients with SAD	DSM-IV Insomnia Severity Index Liebowitz Social Anxiety Scale (LSAS)	Two-thirds of our sample of patients with SAD reported mild to severe insomnia symptoms
Soehner et al., 2012 (Soehner and Harvey, 2012)	N = 5692 NCS-R respondents with no mood or anxiety disorder (n = 3711), mood disorders only (n = 327), anxiety disorders only (n = 1137), and coexisting mood and anxiety disorders (n = 517)	DSM-IV in the past year	Rates of insomnia complains were 34.8 in SAD
	Cross-sectional		
Alvaro et al., 2014 (Alvaro et al., 2014)	N = 318	DSM-IV	**Social phobia was not significantly related to insomnia. Eveningness apredicted the models in which insomnia was predicted by social phobia.**
	South Australian school students cross-sectional study.	Insomnia severity idex	
Steinsbekk et al., 2015 (Steinsbekk and Wichstrøm, 2015)	N = 1250 children 6 years old	DSM-IV	Insomnia increased the risk for developing symptoms of conduct disorder, major depressive disorder, and social phobia
Alvaro et al., 2017 (Alvaro et al., 2017)	N = 318 College students longitudinal	DSM-IV	insomnia symptoms predicted symptoms of depression, and vice-versa. Symptoms of social phobia predicted symptoms of insomnia disorder but not vice-versa.
		Revised Child Anxiety and Depression Scale (RCADS) cronotype	
		Insomnia severity idex (ISI)	
Blumenthal et al., 2019 (Blumenthal et al., 2019)	N = 10,140	DSM-IV	insomnia symptoms were positively related to both SAD and Alcohol use disorder (AUD) status. The current findings do indicate insomnia may be an important target to address in prevention and treatment efforts for SAD, AUDs, and their co-occurrence.
	National Comorbidity Survey-Adolescent Supplement were Participants		
	Retrospective past year		
**Social Anxiety and insomnia therapy**			
Tang et al., 2010 (Tang, 2010)	Single case	sleep diary and questionnaires	The improvement of sleep was accompanied by a decrease in sleep-related anxiety and dysfunctional beliefs and attitudes about sleep. In addition, the patient reported a corresponding improvement in daytime functioning and general anxiety.
	Insomnia comorbid with SAD		
	Received CBT-I		
	9 months follow up		
Mason et al., 2014 (Mason and Harvey, 2014)	N = 266 patients patients with anxiety related disorders	Mini-Social Phobia Inventory (Mini-SPIN)	9.19% of the sample consisted of SAD patients, and 26.9% exhibited insomnia symptoms. Those with insomnia reported more severe anxiety and depression symptoms than those without insomnia. CBT focusing on anxiety and/or depression was associated with reductions in insomnia, anxiety, depression, and disability symptoms, but had no effect on sleep duration.
	Including SAD	Insomnia Severity Index (ISI)	
	Underwent to CBT for anxiety disorders		

Current data show that insomnia is very frequent in patients with SAD interesting from the 26% to the 70% of the patients (Soehner and Harvey, 2012; Mason and Harvey, 2014; Raffray et al., 2011). Insomnia has been related to SAD severity and to the high comorbidity with depression and substance use disorder (Alvaro et al., 2017; Blumenthal et al., 2019). Targeting insomnia in the contest of SAD seems important; otherwise, traditional therapeutic approaches for this disorder do not resolve insomnia symptoms (Mason and Harvey, 2014; Tang, 2010).

Insomnia in SAD is poorly studied, while understanding the interactions between insomnia, SAD and depression is needed to improve treatment strategies in patients with SAD. It could allow adapting cognitive and behavioral therapy for insomnia (CBT-I) programs to social anxiety and may be improving anxiety and depressive symptoms in these patients.

#### 3.2.4 Separation anxiety

Separation anxiety disorder (SA) is characterized by persistent and excessive anxiety associated with separation from the patient's most significant other to an inappropriate extent from the developmental psychological point of view. Its lifetime prevalence is 4.8%. Usually, It is initially diagnosed in childhood, but recent studies have shown that it is increasingly common in adulthood as well (American Psychiatric Association, 2022; Miniati et al., 2012, 2017; Pini et al., 2021, 2023). Limited research exists examining insomnia and separation anxiety. Only 3 studies evaluated insomnia in the context of separation anxiety and regards children (Table 4) (Alvaro et al., 2017; Paine and Gradisar, 2011; Steinsbekk et al., 2013b). It emerged that insomnia may predict symptoms of separation anxiety in preschoolers (Alvaro et al., 2017; Steinsbekk et al., 2013b). In one study, by Pain et al. (2011) (Paine and Gradisar, 2011), the effect of CBT-Insomnia was compared to or waitlist control in a group of children aged 7–13 year with insomnia. Compared to waitlist controls, children receiving CBT-I showed significant improvements in sleep but also in symptoms of separation anxiety. Insomnia in separation anxiety is poorly studied, while insomnia symptoms may play a role in this anxiety disorder. More studies are needed in this field.

**Table 4:** Studies on insomnia symptoms Separation Anxiety (SA).

Separation Anxiety Disorder			
Pain et al., 2011 (Paine and Gradisar, 2011)	N = 42 children (M = 9.3 ± 1.9 yrs, range 7–13 yrs, 18f, 24 m) were randomized to CBT-I (N = 21) or waitlist control (N = 21). CBT-I consisted of 6 sessions	Sleep diary and actighraphic registration	Compared to waitlist controls, children receiving CBT-I showed significant improvements in sleep latency, wake after sleep onset, and sleep efficiency. CBT was also associated with a reduction in problematic sleep associations (p ≤ 0.001), and separation anxiety (p ≤ 0.01), with all gains being maintained 6 months post-treatment
Steinsbekk et al., 2013 (Steinsbekk et al., 2013b)	N = 1250 children children born in 2003 or 2004 in Trondheim, Norway, who attended regular community health checkups for 4 year olds	DSM-IV	primary insomnia,16.6% was specifically related to symptoms of separation anxiety
Alvaro et al., 2017 (Alvaro et al., 2017)	Adelaide, South Australia, at two time-points approximately 6 months apart. The study was completed by 318 and 255 high school students at baseline and follow-up, respectively, aged 12-18	DSM-IV	symptoms of insomnia predicted symptoms of separation anxiety disorder but not vice-versa
		Revised Child Anxiety and Depression Scale (RCADS)	
		cronotype	
		Insomnia Severity Index	

#### 3.2.5 Obsessive-compulsive disorder (OCD)

Chronic and debilitating, obsessive-compulsive disorder (OCD) is defined by intrusive, unwanted, and unpleasant thoughts, obsessions, and ritualized behaviors known as compulsions (American Psychiatric Association, 2022). The goal of compulsions, which may take the form of both overt actions and mental rituals, is to alleviate the mental suffering caused by obsessions. Some studies underline the link between disturbed sleep, poor sleep and OCD (for an overview, see (Steinsbekk et al., 2013b; Cox et al., 2018a; Díaz-Román et al., 2015; Nota et al., 2015; Timpano et al., 2014)). Indeed, existing research on the relationship between OCD and insomnia is scarce and includes 12 studies only according with inclusion/exclusion criteria (Table 5) (Alvaro et al., 2014, 2017; Mason and Harvey, 2014; Raines et al., 2015; Sevilla-Cermeño et al., 2019, 2020; Osnes et al., 2020, 2021; Cox and Olatunji, 2021; Nordahl et al., 2018; Hagen et al., 2021; Wright et al., 2011). In particular, the association between insomnia and OCD started to be considered from 2014.

**Table 5:** Studies on insomnia symptoms and Obsessive Compulsive Disorder OCD.

Obsessive Compulsive Disorder (OCD)			
Timpano et al. (2014) (Raines et al., 2015)	N = 167 young adults at a large university in the United States	Yale-Brown Obsessive Compulsive Scale (YBOCS; G)	Significant correlation between obsessions and symptoms of insomnia, but none between insomnia and compulsions. When investigating the various dimensions, there was a specific relationship between insomnia and obsessions. Despite the fact that depression is frequently highly comorbid with both OCD and sleep disturbances, depressive symptoms did not explain the OCS-sleep relationship in either study, suggesting a unique relationship between obsessions and insomnia. High levels of intrusive thoughts are associated with insomnia symptoms, according to the results.
		Insomnia Severity Index (ISI)	
Alvaro et al., 2014 (Alvaro et al., 2014)	N = 318 high school students from grades South Australian cross-sectional	DSM-IV	After confounder variables OCD was not significantly related to insomnia. Eveningness predicted the models in which insomnia was predicted by OCD
		Insomnia Severity Index (ISI)	
[Bibr bib115][Bibr bib132]	N = 526 individuals recruited through Amazon's Mechanical Turk (Mturk), an online crowdsourcing marketplace	Yale-Brown Obsessive Compulsive Scale (YBOCS; G)	Results revealed distinct associations between the unacceptable thoughts domain of OCD and symptoms of insomnia.
		Insomnia Severity Index (ISI)	
Alvaro et al., 2017 (Alvaro et al., 2017)	N = 318 Students longitudinal	DSM-IV	Symptoms of OCD predicted symptoms of insomnia disorder but not vice-versa.
		Revised Child Anxiety and Depression Scale (RCADS)	
		cronotype	
		Insomnia severity idex	
Sevilla-Cermeño et al., 2019 (Osnes et al., 2020)	N = 193 patients from a specialist pediatric OCD clinic	DSM-5 criteria for OCD	42% (81/193) of the sample showed clinical insomnia. These participants had significantly higher OCD severity, higher rates of psychiatric comorbidities, more severe depressive symptoms, poorer general functioning, and were more likely to take sleep medications, compared to patients without insomnia
	Cross-sectional	Insomnia Severity Index (ISI)	
		Children's Yale-Brown Obsessive-Compulsive Scale (CY-BOCS)	
Osnes et al., 2020 (Sevilla-Cermeño et al., 2020)	n = 1563 large population-based Akershus Birth Cohort data from the hospital's birth records and questionnaire responses from pregnancy weeks 17 and 32 and postpartum week 8 observational	Hopkins Symptom Check List (HSCL)	The observed prevalence of obsessive-compulsive disorder was higher after delivery (4.2%) than during pregnancy (2.5%). Insomnia during pregnancy was substantially associated with postpartum anxiety symptoms, after adjusting for a number of psychosocial and reproductive variables, as determined by multiple regression analysis. This association was significantly diminished, however, when depression variables were included in the analysis, indicating that gestational insomnia may also be a marker for a mood disorder.
		Mini-International Neuropsychiatri Interview (MINI)	
		Bergen Insomnia Scale (BIS)	
Sevilla-Cermeño et al., 2020 (Osnes et al., 2021)	N = 31.856 OCD patients were identified from a cohort of 13,017,902 individuals living in Sweden anytime during 1973 and 2013 longitudinal	ICD-10	Individuals with OCD had almost 7-fold increased odds of receiving an insomnia diagnosis or being dispensed a drug with specific indication for insomnia, compared to unaffected individuals from the general population (42.2% vs. 11.0%, respectively; OR = 6.92 [95% CI, 6.76–7.08]). When individuals with comorbid depression and anxiety disorders were excluded, the odds of insomnia were significantly reduced (OR = 4.97 [95% CI, 4.81–5.14] and OR = 4.51 [95% CI, 4.33–4.69], respective
Osnes et al., 2021 (Cox and Olatunji, 2021)	n = 530 Norwegian Depression and Anxiety in the Perinatal Period (DAPP) prospective, population-based, cohort study. Hospital birth records and questionnaire responses from pregnancy week 17 and postpartum week 8	Hopkins Symptom Check List (HSCL)	Mid-pregnancy insomnia was significantly associated with both concurrent and postpartum anxiety in a linear mixed model adjusted for several potential confounders. Participants with mid-pregnancy insomnia had significantly higher levels of perinatal anxiety and postpartum OCD symptoms than participants with normal mid-pregnancy sleep. OCD symptoms affected more women after delivery than before (6.4% vs. 3.8% p = 0.034).
		Mini-International Neuropsychiatri Interview (MINI)	
		Bergen Insomnia Scale (BIS)	
Cox et al., 2021 (Nordahl et al., 2018)	1, 2020 community adults pre-coronavirus outbreak insomnia symptoms as a predictor of post-coronavirus outbreak OCD symptoms in a sample of community adults N = 369 completed the survey		Significant correlations were observed between pre-coronavirus outbreak insomnia symptoms and post-coronavirus outbreak OCD symptoms. Except for washing and hoarding symptoms, which were unrelated to pre-coronavirus insomnia symptoms, similar results were observed for specific OCD symptom dimensions. During the pandemic, there was no evidence of a reverse effect of prior OCD symptoms on insomnia symptoms. These results suggest that those with symptoms of insomnia prior to the coronavirus pandemic may be susceptible to an increase in certain OCD symptoms during the pandemic.
**Insomnia treatment and OCD**			
Mason et al., 2014 (Mason and Harvey, 2014)	N = 266 patients with anxiety related disorders	Insomnia Severity Index	Patients with DOC were the 11.38% of the sample 30.8 %experienced insomnia symptoms. Individuals with insomnia reported more severe symptoms of anxiety and depression than individuals without insomnia. CBT focused on anxiety and/or depression was associated with reductions in symptoms of insomnia, anxiety, depression, and disability but it was ineffective in improving sleep duration
	Including OCD		
	Underwent to CBT for anxiety disorders		
Nordahl et al., 2018 (Hagen et al., 2021)	N = 36 patients with OCD underwent concentrated exposure treatment delivered in a group over four consecutive days and were assessed at three different time points (pre, post and 6 months follow-up).	M.I.N.I. Yale-Brown Obsessive-Compulsive scale (Y-BOCS; Bergen Insomnia Scale (BIS)	At pre-treatment nearly 70% of the patients reported sleep difficulties indicative of primary insomnia. results showed that patients had large reductions of OCD-symptoms as well as significant improvements in sleep disturbance assessed after treatment, However, a proportion of the patients suffered from residual symptoms of insomnia after treatment.
Hagen et al., 2021 (Wright et al., 2011)	N = 42 patients with OCD	M.I.N.I. Yale-Brown Obsessive-Compulsive scale (Y-BOCS; Bergen Insomnia Scale (BIS)	There were no significant differences in insomnia symptoms between the conditions post-treatment, but patients who received B4DT showed a significant moderate improvement at 3-month follow-up. There was no association between insomnia and OCD treatment outcome, and changes in insomnia symptoms were primarily related to changes in depressive symptoms. The primary conclusion is that concentrated exposure therapy is efficacious regardless of the presence of comorbid insomnia, and that insomnia symptoms are moderately reduced after treatment.
	A recent randomized controlled trial randomized participants to the Bergen 4-day treatment (B4DT, *n* = 16), or 12 weeks of unguided self-help (SH, *n* = 16), or waitlist (WL, *n* = 16).		

Insomnia is now considered risk factors for OCD symptoms, including the perinatal period and the pandemic (Osnes et al., 2021; Cox and Olatunji, 2021). Insomnia is very frequent in OCD patients and may interest 30.8 % to 70% of patients (Table 5). Insomnia was most related to OC symptoms compared to other sleep disturbances such as delayed bedtime and nightmares. This relationship was particularly strong between insomnia and the obsessions and unacceptable thoughts domain of OCD and with the severity of OCD. Similarly to other anxiety disorders therapeutic approach for OCD may improve insomnia symptoms too but a proportion of the patients suffered from residual symptoms of insomnia after treatment. Hence specific insomnia approach has been suggested in OCD (Table 5).

Insomnia symptoms in OCD are poorly studied, while it is emerging an important role in the development and severity of OCD. More studies are needed in this field.

#### 3.2.6 Post Traumatic Stress Disorder

Post-traumatic stress disorder (PTSD) is a common mental illness triggered by experiencing or witnessing traumatic events. Individuals with PTSD or posttraumatic stress symptoms manifest re-experiencing, avoidance, and hyperarousal and reactivity symptoms following exposure to a traumatic event (American Psychiatric Association, 2022). Trauma-exposed populations report higher than average rates of disturbed sleep; in fact, sleep disturbance is often considered a “hallmark” feature of PTSD (American Psychiatric Association, 2022). Comorbidity estimates of insomnia are high, ranging from 39.6 to 81.6%, with nightmares and parasomnia as well as other parasomnias, such as —both insomnia and nightmares—are included as diagnostic criteria for PTSD (American Psychiatric Association, 2022).

Existing research on the relationship between Post Traumatic Stress Disorder (PTSD) and insomnia is wide and 43 studies were included according to inclusion/exclusion criteria (Table 6) [33,51, 53,90-126]. Many other studies have been excluded because the insomnia evaluation was conducted with no standardized measures, such as with one question or an item of another rating scale, or other majority of the studies regarded sleep quality, general sleep disturbances or sleep duration.

**Table 6:** Studies on insomnia symptoms and Posttraumatic Stress Disorder- PTSD.

**Posttraumatic Stress Disorder- PTSD**			
Babson et al., 2008 (Babson et al., 2008)	N = 5692 (3311 females; M(age) = 43.33, SD = 16.55) adults from the National Comorbidity Survey-Replication	DSM-IV	Nicotine dependence was a partial mediator of the relations between insomnia symptoms and both PD and PTSD among a nationally representative sample Overall, results suggest nicotine dependence may be a possible mechanism that underlies insomnia among those with PD and PTSD.
	Cross-sectional	Insomnia Severity Index (ISI)	
Write et al., 2011 (Wright et al., 2011)	*N* = 659 active duty soldiers in a brigade combat team assessed 4 months after their return from a 12-month deployment to Iraq, and then again 8 months later Longitudinal	Insomnia Severity Index (ISI)	Although insomnia and psychological symptoms were associated at both time periods and across time periods, insomnia at 4 months post deployment was a significant predictor of change in depression and PTSD symptoms at 12 months post deployment, but depression and PTSD symptoms at 4 months post deployment were not significant predictors of change in insomnia at 12 months post deployment.
Nadorff et al., 2011 (Nadorff et al., 2011)	N = 583 undergraduate students at a large, public university in southeastern United States	The PTSD Checklist-Civilian Version (PCL)	The relation between insomnia and suicidal ideation is mediated by symptoms of depression, anxiety, and PTSD.
		Insomnia Severity Index (ISI)	
Soehner et al., 2012 (Soehner and Harvey, 2012)	N = 5692 NCS-R respondents with no mood or anxiety disorder (n = 3711), mood disorders only (n = 327), anxiety disorders only (n = 1137), and coexisting mood and anxiety disorders (n = 517)	DSM-IV criteria in the past year	Rates of insomnia were 16.6 in patients with PTSD.
	Cross-sectional		
Krakow et al., 2012 (Krakow et al., 2012)	N = 1078 patients seeking care at a sleep medical center		Chart review of the patients presented with one of three sleep complaints, poor sleep quality, 51%; sleep-disordered breathing, 26%; and insomnia, 24%, and 32% reported moderate to severe Post Traumatic Stress. Both insomnia and time monitoring severity were greater in the 350 patients with Post Traumatic Stress compared with the 728 patients with minimal or no such symptoms.
	Retrospettive study		
Hughes et al., 2013 (Hughes et al., 2013)	N = 107 women Veterans with insomnia (mean age = 49 years, 44% non-Hispanic white),	Insomnia Severity Index (ISI)	55% exhibited probable symptoms of post-traumatic stress disorder (PTSD). There was a correlation between probable PTSD and more severe self-reported sleep disruption and psychological distress. Higher PCL-C total score was a significant independent predictor of worse insomnia severity index score in a regression model, whereas other factors were not.
		Posttraumatic Stress Disorder PTSD Checklist-Civilian (PCL-C)	
Hall Brown et al., 2015 (Hall Brown et al., 2015)	N = 554 nonclinical, urban, young adult African Americans was recruited for a larger study	DSM-IV	40% (n = 185) of the sample screening positively for mild to severe insomnia. Ninety-seven percent (n = 450) of the sample endorsed exposure to at least one trauma (probable event) during their lifetime, with 90% (n = 417) endorsing exposure to at least one traumatic experience before the age of 16 y Insomnia was significantly associated with sexual trauma (Chi-square [1, 465] = 39.38, p < 0.001), physical assault (Chi-square [1, 465] = 7.50, p < 0.01), accidents (Chi-square [1465] = 8.72, p < 0.01), natural disasters (Chi-square [1, 465] = 5.43, p < 0.05), and exposure to sudden violent death (Chi-square [1, 465] = 22.71, p < 0.001) compared to never having been exposed to these types of traumas. The remaining included trauma categories, fire/explosions and life threatening illness/injury, were not significantly associated with insomnia.
	Retrospective	Insomnia Severity Index (ISI)	
		Posttraumatic Stress Disorder Checklist (PCL)	
Jenkins et al., 2015 (Jenkins et al., 2015)	N = 917 newly enrolling Operation Iraqi Freedom/Operation Enduring Freedom/Operation New Dawn (OEF/OIF/OND) veterans VA San Diego Healthcare System (VASDHS) between March 2012 and August 2013	PTSD Checklist-Civilian Version (PCL-C), Insomnia Severity Index (ISI)	3.1% of veterans without Military sexual trauma and 60.8% of veterans with Miltary sexual trauma had clinically significant insomnia symptoms, with the MST subsample reporting more severe symptoms, P < 0.05. Insomnia was more prevalent than depression, hypomania, PTSD, and substance misuse. Veterans with insomnia were more likely to seek care for depression and PTSD symtpoms <0.001.
Brownlow et al., 2017 (Brownlow et al., 2017)	N = 21,449	Brief Insomnia Questionnaire	Military soldiers with current major depressive episode (MDE) had the highest prevalence of insomnia disorder (85.0%), followed by current generalized anxiety disorder (GAD; 82.6%) and current posttraumatic stress disorder (PTSD; 69.7%), respectively. Significant interactions were found between insomnia and psychiatric disorders; specifically, MDE, PTSD, and GAD status influenced the relationship between insomnia and memory/concentration problems.
	Army Study to Assess Risk and Resilience in Service members (STARRS) U.S. Army soldiers	PTSD Checklist for DSM-5 (PCL-5)	
Psarros et al., 2017 (Psarros et al., 2017)	N = 92	ICD-10	The presence of insomnia was identified in 63.0% of the victims. 46.7% of the participants were diagnosed with PTSD in the first post-disaster month, while 51.1% of the total sample experienced ‘fear of imminent death’. **The majority of sleep complaints were significantly more common among PTSD patients. Insomnia was independently associated with female gender, PTSD, advanced age, and “fear of impending death.” The findings of the present study indicate that female victims of wildfires who experienced ‘fear of imminent mortality’ and developed PTSD were more likely to be diagnosed with insomnia and to report specific insomnia symptoms.**
	Randomly chosen victims of wildfires in the Greek province of Ilia	Athens Insomnia Scale (AIS)	
	Retrospective		
Cox et al., 2018 (Cox et al., 2018b)	N = 72 combat exposed veterans	PTSD Checklist	**There are associations between insomnia symptoms and PTSD symptom clusters, as well as a significant interaction between insomnia symptoms and combat exposure that predicts reexperiencing but not avoidance or arousal symptoms of PTSD.**
		Insomnia Severity Index (ISI)	
Han et al., 2018 (Han et al., 2018)	N = 43 females who reported having a history of sexual violence (mean age 26.56 ± 7.81) retrospective	Insomnia Severity Index, ISI	53.7% reported insomnia symptoms. Results indicate that higher severity of insomnia was associated with more PTSD symptoms, more trauma-related distress, and less perception of trauma-related guilt
		PTSD Symptom Scale Self-Report	
Short et al., 2018 (Short et al., 2018)	N = 30 individuals with PTSD	PTSD-specific symptoms	Multi-level modelling analyses indicated that, after accounting for baseline PTSD symptom severity, PTSD-specific factors were associated with insomnia symptoms, but insomnia-specific factors were not. Only daytime PTSD symptoms and fear of sleep predicted nightmares. Both sleep- and PTSD-related factors play a role in maintaining insomnia among those with PTSD, while nightmares seem to be linked more closely with only PTSD-related factors.
		Insomnia Severity Index, ISI	
Hansen et al., 2018 (Hansen et al., 2018)	N = 438	Primary Care-Post-Traumatic Stress Disorder Screen	16.4% screened positive for moderate or even severe levels of clinical insomnia. Bivariate correlations demonstrated that sleep problems were correlated with PTSD symptoms (r = 0.41, p < 0.001), depression (r = 0.49, p < 0.001), Sleep Hygiene Routine (r = −0.34, p < 0.001), and more frequent use of multiple devices before bed (r = 0.15, p = 0.002).
	Army National Guard sample	Insomnia Severity Index ISI	
	Cross sectional		
Belleville et al., 2019 (Belleville et al., 2019)	N = 379 evacuees	Insomnia Severity Index (ISI)	The interview confirmed that 29.1% met criteria for PTSD, 25.5% for depression, and 43.6% for insomnia; in most cases, insomnia was related to the fires. The most frequently reported post-traumatic stress symptoms were repeated disturbing memories (reported by 77.4% of the sample), feeling upset when reminded of the stressful experience (76.7%), and trouble falling or staying asleep (72.5%)
	May 2016 wildfires in Fort McMurray (Alberta, Canada).	PTSD Checklist for DSM-5 (PCL-5)	
Grossmann et al., 2020 (Grossman et al., 2019)	N = 108	ICD-11 PTSD	**Complex PTSD was prevalent (**>**50%) and correlated strongly with insomnia. 95% of those diagnosed with Complex PTSD suffered from insomnia. The Complex PTSD group was 18 times more likely to experience insomnia than the control group.**
	The genocide of Yazidis	Insomnia Severity Index (ISI)	
	Yazidi women former captives of the Islamic State terrorist group		
Angehrn et al., 2020 (Angehrn et al., 2020)	*n* = 8520 public safety personnel (PSP)	Insomnia Severity Index (ISI)	Many PSP in each category reported symptoms consistent with clinical insomnia (49–60%). Insomnia symptoms was correlated with PTSD, depression, anxiety, and alcohol use disorder for all PSP categories (*r* = 0.18–0.70, *p* < 0.001). PSP who screened positive for insomnia were 3.43–6.96 times more likely to screen positive for a mental disorder
	Cross sectional	PTSD Checklist for DSM-5 (PCL-5)	
Blekas et al., 2020 (Blekas et al., 2020)	N = 270	Posttraumatic Stress Disorder–8	Criteria for a probable posttraumatic stress disorder diagnosis were met by a total of 16.7% (21.7% of women; 5.1% of men). Those suffering with higher levels of PTSD scored positively for insomnia and exhibited significantly higher peritraumatic distress.
	Greek health care professionals during the COVID-19 pandemic.	Athens Insomnia Scale (AIS)	
	Cross sectional		
Colvonen et al., 2020 (Colvonen et al., 2019)	N = 5552 post-9/11 veterans observational study	PCL-5 (PTSD Checklist–DSM-5	Approximately 57.2% of the sample had insomnia symptoms. The cohort was also at a high risk for a variety of clinical disorders, including PTSD, which was associated with higher rates of insomnia symptoms (93.3%).
		Insomnia Severity Index (ISI)	
Gaffey et al., 2020 (Gaffey et al., 2020)	N = 1109 participants in the Women Veterans Cohort Study	PCL-5 (PTSD Checklist–DSM-5) Insomnia Severity Index	PTSD symptom severity was associated with hypertension (r = 0.09, P < 0.001). PTSD symptom severity and hypertension were each associated with the insomnia symptoms difficulty falling asleep, difficulty staying asleep, and worry/distress about sleep problems (PTSD: rs = 0.58–0.62, P < 0.001; hypertension: rs = 0.07–0.10, P < 0.001). Insomnia symptoms mediated the association between PTSD and hypertension.
Geng et al., 2021 (Geng et al., 2021a)	N = 7218 adults recruited from Jiangxi and Hunan provinces in China,	Youth Self-Rating Insomnia Scale (YSIS)	Approximately 67.1% of participants reported one traumatic event; The prevalence of PTSD was 2.1% in the total sample and 3.1% among the trauma-exposed. Among participants with PTSD, 53.6% were screened as depression, 54.3% had insomnia, 65.6%
		PCL-5 (PTSD Checklist–DSM-5)	
Geng, Zou et al., 2021 (Geng et al., 2021b)	N = 7245 adults in China	Rating Insomnia Scale (YSIS)	After adjustment of demographics, Adverse Childhood Experiences ACEs (β = 0.11, p < 0.001) was associated with adulthood insomnia. After controlling for demographics, PTSD and depressive symptoms mediated the relationship between ACEs and insomnia.
		PCL-5 (PTSD Checklist–DSM-5)	
Lu et al., 2021 (Lu et al., 2021)	N = 500 COVID-19 frontline healthcare workers in Taiwan cross-sectional study.	Insomnia Severity Index (ISI)	Prevalence rate of 15.4% for PTSD symptoms, 44.6% for insomnia, 25.6% for depressive symptoms, 30.6% for anxiety symptoms, and 23.4% for stress among the participants. There were significantly positive interrelationships between all these variables. Anxiety symptoms and fear of COVID-19 predicted PTSD whereas symptoms of anxiety, fear of COVID-19, and stress predicted insomnia.
		Depression, Anxiety, and Stress Scale-21	
Mahmoudi et al., 2021 (Mahmoudi et al., 2021)	N = 844, people who had recovered from COVID-19 were called and interviewed	PCL-5 (PTSD Checklist–DSM-5	Insomnia, PTSD, and COVID-19- related self- stigma displayed significant direct associations (r = .334 to 0.454; p < 0.01).
	A cross- sectional design	Insomnia Severity Index (ISI)	
Martínez-Caballero et al., 2021 (Martínez-Caballero et al., 2021)	N = 317 workers	General Health Questionnaire-12 (GHQ-12), the Davidson Trauma Scale (DTS-8), and the Athens Insomnia Scale (AIS-8).	36% of respondents had psychological distress, 30.9% potentially had PTSD, and 60.9% experienced insomnia.
	Emergency Medical Services workers		
	Cross sectional		
Mei et al., 2021 (Mei et al., 2021)	N = 516 medical staff	PTSD Checklist-Civilian, Perceived Stress Scale, Insomnia Severity Index	The findings revealed that 10.5% of the medical staff experienced PTSD symptoms, and that the severity of insomnia mediated the effect of perceived stress on PTSD. In addition, compassion fatigue moderated the relationship between perceived stress and post-traumatic stress disorder (PTSD)
Davis et al., 2022 (Davis et al., 2022)	N = 1230 post-9/11 veterans assessed over four time points across 12 months	PTSD Checklist for DSM-5 (PCL-5)	Higher prior levels of both PTSD and alcohol use were associated with greater worse insomnia symptoms. Worsened insomnia symptoms (was a mechanism linking PTSD and more frequent drinking.
		Insomnia Severity Index	
Carlson et al., 2023 (Carlson et al., 2023)	N = 26,443 current women service members from the Millennium Cohort Study, two waves of survey data (2011–2013, Time 1 [T1] and 2014–2016, Time 2 [T2]) from	Assessed recent traumas in the past 3 years, and probable insomnia at T1 and probable post-traumatic stress disorder (PTSD) and depression at T2. A longitudinal mediation model	Women who had experienced recent sexual assault (odds ratio [OR] = 1.68; 95% CI = 1.24–2.10), sexual harassment (OR = 1.22; 95% CI = 1.05–1.41), and combat (OR = 1.34; 95% CI = 1.20–1.49) at T1 had a greater risk of probable insomnia at T1 compared with women who had not recently experienced these events. Probable insomnia at T1, in turn, was associated with probable depression (OR = 2.66; 95% CI = 2.31–3.06) and PTSD (OR = 2.57; 95% CI = 2.27–2.90) at T2. Insomnia contributes to the risk of subsequent mental health conditions following trauma.
**Insomnia treatment in PTSD**			
Zayfert et al., 2004 (Zayfert and DeViva, 2004)	N = 27 patients who no longer met PTSD diagnosis and underwent cognitive behavioral therapy (CBT) for PTSD A retrospective analysis of posttreatment	Clinician-Administered PTSD Scale (CAPS)	**According to the data, 48% reported persistent insomnia. Insomnia persisted for the vast majority despite the absence of recurring nightmares and hypervigilance. These findings suggest that interventions to resolve factors contributing to PTSD-related insomnia should be studied.**
		Insomnia Severity Index	
Swanson et al., 2009 (Swanson et al., 2009)	N = 10 combat veterans participated in a 10-session group treatment combining cognitive-behavioral therapy for insomnia with exposure, rescripting, and relaxation therapy for nightmares	Posttraumatic Diagnostic Scale	Participants reported improvements in sleep and nightmares following treatment.
		Insomnia Severity Index	
Margolies et al., 2011 (Margolies et al., 2013)	N = 40 combat veterans (mean age 37.7 years; 90% male and 60% African American) who served in Afghanistan and/or Iraq (Operation Enduring Freedom [OEF]/Operation Iraqi Freedom [OIF]) to 4 sessions A combined intervention comprised of CBT-I (Cognitive Behavioral therapy for Insomnia) combined with imagery rehearsal therapy (IRT) or wait list control	Insomnia Severity Index	The findings indicate that an intervention targeting trauma-specific sleep disturbance produces significant short-term effects, including significant reductions in PTSD symptoms and the severity of insomnia.
Krakow et al., 2012 (Krakow et al., 2012)	N = 62 PTSD patients	Sleep Impairment Index	At post-treatment, veterans who participated in CBT-I/IRT reported improved subjectively and objectively measured sleep, a reduction in PTSD symptom severity and PTSD-related nighttime symptoms, and a reduction in depression and distressed mood compared to the waitlist control group. The findings suggest that CBT-I combined with adjunctive IRT may hold promise for reducing both insomnia and PTSD symptoms.
	University of New Mexico Health Sciences Center Participants were recruited from rape crisis centers or crime victims advocacy groups	Posttraumatic Stress Diagnostic Scale	
	Longitudnal study 3-month follow-up		
Talbot et al., 2014 (Talbot et al., 2014)	N = 45 Randomized controlled trial with two arms: CBT-I and monitor-only waitlist control.	Structured Clinical Interview for DSM-IV (SCID), the Clinician-Administered PTSD Scale (CAPS), a portion of the Duke Structured Interview for Sleep Disorders (DSISD),	Successful treatment for insomnia and nightmares in crime victims was associated with improvement in symptoms of PTSD, anxiety, and depression.
	Department of Veterans Affairs (VA) Medical Center adults with PTSD and insomnia, randomly assigned to CBT-I or monitor-only waitlist control		
Ulmer et al., 2018 (Ulmer et al., 2018)	N = 22 veterans with PTSD and insomnia	DSM-IV-R Insomnia Severity Index	CBT-I was superior to the waitlist control condition in all sleep diary outcomes and in polysomnography-measured total sleep time. Compared to waitlist participants, CBT-I participants reported improved subjective sleep (41% full remission versus 0%), disruptive nocturnal behaviors and overall work and interpersonal functioning. These effects were maintained at 6-months follow-up. Both CBT-I and waitlist control participants reported reductions in PTSD symptoms and CAPS-measured nightmares. Cognitive behavioral therapy for insomnia (CBT-I) improved sleep in individuals with posttraumatic stress disorder, with durable gains at 6 months
	A combined intervention comprised of CBT-I (Cognitive Behavioral therapy for Insomnia) and imagery rehearsal therapy (IRT) was evaluated against a usual care comparison group.		
Kanady et al., 2018 (Kanady et al., 2018)	N = 45 adults with PTSD and insomnia. Participants were randomly assigned to 8 weeks of Cognitive behavioral therapy for insomnia (CBT-I) (n = 29) or a waitlist control condition (n = 16).	Fear of Sleep Inventory	Greater fear of sleep was associated with more severe PTSD symptoms, more frequent nightmares, and more intense hypervigilance. Greater sleep fear was associated with decreased wake after sleep onset, decreased total sleep time, and increased disruptive nocturnal behaviors. Following CBT-I, sleep phobia decreased significantly compared to the waitlist condition. These enhancements remained six months later. Fear of sleep was associated with trauma-specific sleep disturbances rather than “classic” insomnia symptoms. Fear of sleep decreased as a result of CBT-I, despite not being a permissible objective for this research protocol and not being associated with insomnia symptoms.
		Clinician Administered PTSD Scale	
		Insomnia Severity Index	
		polysomnography, sleep diaries	
Colvonen et al., 2019 (Colvonen et al., 2019)	N = 40 veterans with comorbid PTSD and Substance Use Disorder (SUD) in a 28-day Substance Abuse Residential Rehabilitation Treatment Program (SARRTP) PTSD track. baselineposttreatment, 3-month follow-up	Clinician Administered PTSD Scale	Results of the longitudinal mixed model showed that PTSD symptoms improved over time but that insomnia symptoms did not. Individuals with greater insomnia severity at the start of treatment had more severe baseline PTSD symptomatology. These findings are consistent with the PTSD outpatient treatment findings and further adds evidence that insomnia is unremitting without direct intervention. Given the relationship insomnia has with PTSD severity, SUD, and relapse, directly targeting insomnia may further help improve.
		Insomnia Severity Index	
[Bibr bib157][Bibr bib157]	Veterans N = 165	20-item PTSD Checklist for DSM-5 (PCL-5)	Most veterans reported at least moderate difficulties with insomnia at both pretreatment (83.0%–95.1%) and posttreatment (69.1–71.3%). Statistically significant reductions in self-reported insomnia severity occurred from pretreatment to posttreatment; Longitudinal mixed-effects models showed a significant interactive effect of Changes in Insomnia × Time in predicting PTSD and depression symptoms, indicating that patients with more improvements in insomnia had more positive treatment outcomes. These findings suggest that many veterans continued to struggle with sleep disruption after a 3-week ITP, and successful efforts to improve sleep could lead to better PTSD treatment outcomes. Further research is needed to establish how adjunctive sleep interventions can be used to maximize both sleep and PTSD outcomes.
	3-week intensive treatment program (ITP) for veterans with PTSD that integrated cognitive processing therapy (CPT), mindfulness, yoga, and other ancillary services	Insomnia Severity Index	
Kaczkurkin et al., 2021 (Kaczkurkin et al., 2021)	N = 326 patients seeking treatment at a clinic specializing in CBT for anxiety	DSM-5	Insomnia was significantly associated with PTSD, GAD, and panic disorder after controlling for the overlap between classes of anxiety symptoms and depressive symptoms. Insomnia may persist after treatment for anxiety, suggesting that CBT for insomnia may be necessary during or after a course of CBT for anxiety.
		Insomnia Severity Index	
		Posttraumatic Diagnostic Scale for DSM-5 (PDS-5)	
Pigeon et al., 2021 Pigeon et al. (2022)	N = 110 participants with PTSD and insomnia a 20-week trial, were randomized to Cognitive behavioral therapy for insomnia (CBT-I) followed by CPT or to attention control followed by CPT. Primary outcomes following CBT-I (or control) were the 6-week change	Clinician-administered PTSD scale (CAPS)	At 6 weeks, the CBTi condition had greater reductions in ISI, HAM-D, and CAPS scores than the attention control condition. At 20 weeks, participants in the CBTi + CPT condition had greater reductions in ISI, HAM-D, and CAPS scores compared to control + CPT. Effects were larger for insomnia and for depression than for PTSD. Similar patterns were observed with respect to clinical response and remission.
		Insomnia Severity Index	
		Hamilton Rating Scale for Depression (HAM-D).	
Kartal et al., 2021 (Kartal et al., 2021)	N = 693 Australian veterans (ex-serving members of the Australian Defence Force) who participated in an accredited PTSD trauma recovery program	Insomnia Severity Index	Cross-lagged pathway analyses revealed at most time points significant bidirectional pathways between insomnia symptoms and PTSD symptoms. When insomnia symptoms persist following treatment, a sleep-focused intervention and a sequenced approach to treatment are indicated.
	Time points intake, discharge, 3-month, and 9-months posttreatment follow-up.		
Tanev et al., 2022 (Tanev et al., 2022)	N = 326 veterans 9/11 attack 2-week intensive program for PTSD	Insomnia Severity Index	At pretreatment, 73.9% of participants (n = 241) met the criteria for moderate or severe insomnia, whereas at posttreatment 67.7% of participants (n = 203) met the criteria. Analyses revealed that posttreatment hyperarousal symptoms were associated with posttreatment insomnia. These findings suggest that although an intensive program for service members and veterans with PTSD may significantly reduce insomnia symptoms, clinically meaningful residual insomnia symptoms remain.
	N = 326 veterans 9/11 attack 2-week intensive program for PTSD		
Ranney et al., 2022 (Ranney et al., 2022)	N = 91 military veterans with insomnia disorder randomized to Brief behavioral treatment for insomnia (BBTI, 4 sessions) or progressive muscle relaxation therapy	Insomnia Severity Index	In contrast to progressive muscle relaxation therapy, BBTI significantly reduced trauma-related nightmares from baseline to posttreatment.

Data showed that insomnia may predict subsequent PTSD (Table 6) (Soehner and Harvey, 2012; Wright et al., 2011). A recent meta-analysis of 75 studies estimates that the prevalence of insomnia in PTSD was 63% [CI: 45%–78%]. A medium-size significant correlation was found [ES: 0.52 (CI: 0.47–0.57)] with moderating effects of the COVID-19 pandemic and military service as causes of trauma (Ahmadi et al., 2022).

Insomnia may contribute to maladaptive stress and emotional responses in PTSD and may complicate its trajectory, increasing the risk of depression, substance use disorder or suicidal risk (Table 5) (Babson et al., 2008; Nadorff et al., 2011; Krakow et al., 2012; Hughes et al., 2013; Hall Brown et al., 2015; Jenkins et al., 2015; Brownlow et al., 2017; Psarros et al., 2017; Cox et al., 2018b; Han et al., 2018; Short et al., 2018; Hansen et al., 2018; Belleville et al., 2019; Grossman et al., 2019; Angehrn et al., 2020; Blekas et al., 2020; Colvonen et al., 2019; Gaffey et al., 2020; Geng et al., 2021a; Geng et al., 2021b; Lu et al., 2021; Mahmoudi et al., 2021; Martínez-Caballero et al., 2021; Mei et al., 2021; Davis et al., 2022; Carlson et al., 2023).

Standard PTSD treatments may decrease insomnia symptoms, but residual insomnia is frequent, and it is important to specifically target insomnia in PTSD. On the other hand, finding demonstrates that an intervention targeting trauma-specific insomnia produces large short-term effects, including substantial reductions in PTSD symptoms and insomnia severity (Zayfert and DeViva, 2004; Swanson et al., 2009; Margolies et al., 2013; Talbot et al., 2014; Ulmer et al., 2018; Kanady et al., 2018; Zalta et al., 2020; Pigeon et al., 2022; Kartal et al., 2021; Tanev et al., 2022; Ranney et al., 2022).

CBT for insomnia, CBT for insomnia combined with imagery rehearsal therapy or eszopiclone as adjunct therapies resulted in the most effective treatments for insomnia in PTSD (Table 5). (Zayfert and DeViva, 2004; Swanson et al., 2009; Margolies et al., 2013; Talbot et al., 2014; Ulmer et al., 2018; Kanady et al., 2018; Zalta et al., 2020; Pigeon et al., 2022; Kartal et al., 2021; Tanev et al., 2022; Ranney et al., 2022). Data on pharmacological treatments in PTSD regard poor sleep quality or disturbed sleep (for an overview see Colvonen et al., 2018) (Colvonen et al., 2018). Certainly, in the absence of randomized controlled studies, reports and expert opinion have been constructed. Clinical research should evaluate existing pharmacologic drugs in comparative trials or in conjunction with CBT or alternative therapies to assess both short- and long-term sleep results in this group, given the complexity and multifaceted aetiology of insomnia in PTSD.

## 4 Potential mechanisms of a complex link

Anxiety disorders are complex disorders with genetic, epigenetic, and psychological mechanisms contributing to them (Schiele and Domschke, 2018) with various neuroendocrine, neurotransmitter, and neuroanatomical alterations involved (Schiele and Domschke, 2018).

### 4.1 Genetics and epigenetics

Genetic vulnerability for anxiety and insomnia may overlap, indeed, it is emerging evidence for insomnia-specific polygenic effect on anxiety vulnerability (Palagini et al., 2023c). In particular, in recent Mendelian randomization analyses of a large Genomics Consortium, genetically determined insomnia was found to play a causal role in anxiety and PTSD (for an overview see Palagini et al., 2023) (Palagini et al., 2023c). In this framework, sleep reactivity which is characterized as the trait-like degree to which exposure to stress interferes with sleep, has also related to the vulnerability to anxiety (Palagini et al., 2023c; Kalmbach et al., 2016; Palagini et al., 2016; Okajima and Kadotani, 2023). It has correlated to anxiety sensitivity such as fear of negative consequences associated with anxiety arousal identified as a trait vulnerability to anxiety disorders. It is possible that insomnia-vulnerability to stress may contribute to maladaptive stress response and impaired resilience contributing to the development of anxiety (Palagini et al., 2023c; Kalmbach et al., 2016; Palagini et al., 2016; Okajima and Kadotani, 2023; Reffi et al., 2023).

### 4.2 Neuroanatomical circuitry

**Neuroanatomical alterations** involved in insomnia may contribute to anxiety and anxiety-related disorders. Neuroanatomical circuitry of anxiety and related disorders involves hyperactivation of amygdala and insula and hypoactivation of the medial prefrontal cortex (mPFC) (Schiele and Domschke, 2018). In anxiety-related disorders, dysregulation of emotion may reflect a maladaptive expression of fearful and anxious responses, possibly due to deficits in regulation of prefrontal-cortex-amygdala networks (Schiele and Domschke, 2018). In particular, the amygdala, which is responsible for the expression of fear as well as species-specific defensive behaviors, the formation and retrieval of emotional and fear-related memories, lose the control of pre-frontal cortex in anxiety and related disorders (Schiele and Domschke, 2018). In this framework, accumulating neuroanatomical findings suggest that there is likely an overlap between circuitries of sleep regulation and anxiety.

While sleep is critical to the development of these neuroanatomical areas (Uccella et al., 2023; Aquino et al., 2023), for their plasticity and synaptic balance during adult life, insomnia has been related to a maladaptive neuroplasticity in both neurodevelopment and adult life. This may lead to an impairment of emotional, immune and endocrine pathways [4-6-7-9-10-135, 136,137].

Similarly to anxiety, insomnia is characterized by hyperarousal, such as the hyperactivation of stress and inflammatory systems which may impair the regulation of prefrontal-cortex-amygdala networks with a functional impairment of the top-down regulation of emotions (Riemann et al., 2022; Aquino et al., 2023; Riemann et al., 2023b). Insomnia may likely have an anxiogenic impact on brain circuitry, impairing emotion dysregulation and stress response (Van Someren, 2021). REM sleep dysregulation and its consequences on emotion and cognition, may be the pathway linking insomnia and the vulnerability to mental disorders and it may include anxiety disorders (Van Someren, 2021; Riemann et al., 2023b). Insomnia may also have deleterious effects on cognitive functions, with reduced ability to modulate anxious emotions (Palagini et al., 2023a; Riemann et al., 2023b). Insomnia may impair adaptive memory functions useful for fear extinction retention and safety learning hence promoting or maintains anxiety/fear emotions (Palagini et al., 2023a; Riemann et al., 2023b).

In summary, insomnia may dysregulate corticolimbic brain circuits and may amplify the activity of the “fear network” with an anxiogenic effect (Chellappa and Aeschbach, 2022). The effects of sleep disruption on the cerebral correlates underlying anxiety are mostly established from some neuroimaging studies in individuals experiencing insomnia *and* GAD (Saletu et al., 1994) (Table 1). Insomnia may contribute to maladaptive stress response, emotion dysregulation and impaired resilience hence contributing to anxiety (Pace-Schott et al., 2017; Pace-Schott et al., 2023). In particular, insomnia and sleep loss may impair extinction learning, contributing to the aetiology and perpetuation of multiple anxiety disorders (Pace-Schott et al., 2017; Pace-Schott et al., 2023). While sleep is critical to the development of executive function during neurodevelopment poor sleep is theorized to contribute to impaired behavioral and emotional inhibition and increased difficulty refraining from obsessions and compulsions through and day and evening. Research in adults suggests that impaired inhibition following shortened sleep is a potential mechanism by which sleep is linked to anxiety (Palagini et al., 2018).

This is of important since insomnia with short sleep duration is a particular insomnia phenotype which can be frequent (Cox et al., 2020). In complex, sleep disruption may contribute to the aetiology of PTSD by interfering with consolidation in low-level emotion-regulatory memory systems. This deficit may, in part, result from alterations of sleep that interfere with their consolidation, such as REM fragmentation, that have also been found to presage later PTSD symptoms (Pace-Schott et al., 2017; Pace-Schott et al., 2023). In addition, PTSD patients display reduced vagal activity. Vagal activity contributes to the strengthening of memories, including fear extinction memory, and recent studies show that the role of vagus in memory processing extends to memory consolidation during sleep. Pace-Scott et al. showed that disrupted REM may contribute to fear-related disorders by impairing the consolidation of extinction memory also via REM vagal control (Mäder et al., 2023; Yuksel et al., 2023).

### 4.3 Neuromodulators

Different neuromodulators of sleep regulation and/or anxiety have been hypothesized and may be targets for pharmacotherapy of insomnia in anxiety (for an overview, see Chellappa et al., 2022) (Chellappa and Aeschbach, 2022).

In this framework, orexin dysfunction has been recently hypothesized to be implicated in the hyperactivation of arousal-promoting systems in insomnia as well as in stress response and anxiety pathophysiology (Tang et al., 2017). Orexin A and orexin B (also referred to as hypocretin 1 and hypocretin 2, respectively) are a pair of neuropeptides derived from preproorexin. Orexin-producing neurons are located in the lateral hypothalamic area and posterior hypothalamus and the orexinergic fibers are distributed through the entire brain. Orexinergic neurons receive a variety of signals related to environment and involved in the maintenance of arousal. In the mean, while Orexinergic neurons receive innervations from the limbic system, which may mediate emotional arousal and fear-related responses. On the other hand, orexins project broadly to the entire central nervous system and exhibit an excitatory influence on arousal-related neurons. Accordingly, orexinergic system orchestrates various aspects such as sleep and arousal, behavioral and neuroendocrine responses to stress, emotional reactivity, reward‐seeking behaviors, energetic homeostasis, learning and memory (Sakurai et al., 2010; Giardino and de Lecea, 2014).

Data such as these support the hypothesis that insomnia can be due to an inability of the brain to switch off wake-promoting systems such as the orexin system, as well as an inability to switch on sleep-promoting circuits with an instability of the flip-flop switch system (Palagini et al., 2023a; Plante et al., 2012; Palagini and Bianchini, 2022; Tang et al., 2017).

Although orexinergic neuronal projections are present throughout the brain, they are particularly dense in areas of the brain that contribute to different components of anxiety, including the stress and arousal systems with projection to the medial prefrontal cortex. It is possible that orexin dysregulation overlaps with insomnia, anxiety, and anxiety-related disorders. This hypothesis implies that stressful stimuli would increase the activity on orexin neurons, which in turn would release more orexin in brain limbic regions that regulate neural circuits implicated in the expression and extinction of fear memories, emotion regulation and stress response (Sargin, 2019). In humans, individuals with insomnia or anxiety disorders showed high orexin levels compared with controls suggesting a possible hyperactivity state of the orexin system (Johnson et al., 2012; Palagini et al., 2023d). It is possible that orexin may play a critical role in insomnia arousal-based-anxiety and suggest that orexin antagonism may be an effective strategy for reducing chronic hyper-arousal and disrupting the negative reinforcement not only of insomnia but also the cycle of anxiety. Fear extinction is hindered by orexin activation of an infralimbic-amygdala circuit, but enhanced by DORA therapy. Behaviors that avoid danger are facilitated by elevated orexin transmission in the amygdala-cortical-hippocampal circuit. Reduced fear expression occurs in orexin receptor knockout mice, suggesting a pivotal involvement for orexin receptors in this circuit. DORAs, on the other hand, prevent the onset and expression of fear, reducing fear-conditioned startle responses via blocking orexin receptors (149).

In this framework, targeting insomnia with DORAs may play a role not only in insomnia but also in anxiety and related disorders (Han et al., 2020).

Similarly, a dyfunctional GABAergic neurotransmission has been hypothesized in anxiety and insomnia as well (Palagini and Bianchini, 2022) (Nuss, 2015). GABAergic activity from the ventrolateral preoptic nucleus (VLPO) of the hypothalamus exerts an inhibitory control on the ascending arousal network, and activation of GABA-A receptors is thought to be involved in the regulation of sleep. The amygdala's circuitry is controlled, both physiologically and pathologically, by GABAergic neurons and other neurotransmitters. By inhibiting activity in the hypothalamic-pituitary-adrenal axis, GABAergic neurons play a crucial role in the body's reaction to stress. Acute and chronic stress, anxiety disorders, and acute and chronic insomnia have all been linked to low levels of GABA or impaired GABAergic (Biggio et al., 1990). In this context, the deficiency of GABAergic synapses associated with cortical hyperarousal in insomnia has been linked with the hyperactivity of the HPA axis in insomnia (Plante et al., 2012). Maladaptive changes in neuroplasticity are hypothesized in insomnia, reflecting GABAergic alterations, it may contribute to anxiety-related disorders (Palagini and Bianchini, 2022).

In addition, anxiety and related disorders have been increasingly associated with inflammation. A recent meta-analysis (Renna et al., 2018) showed that patients diagnosed with anxiety and related disorders, excluding any patient with a chronic physical illness, compared to healthy controls had moderately higher concentrations of pro-inflammatory markers.

**Fig. 2:**
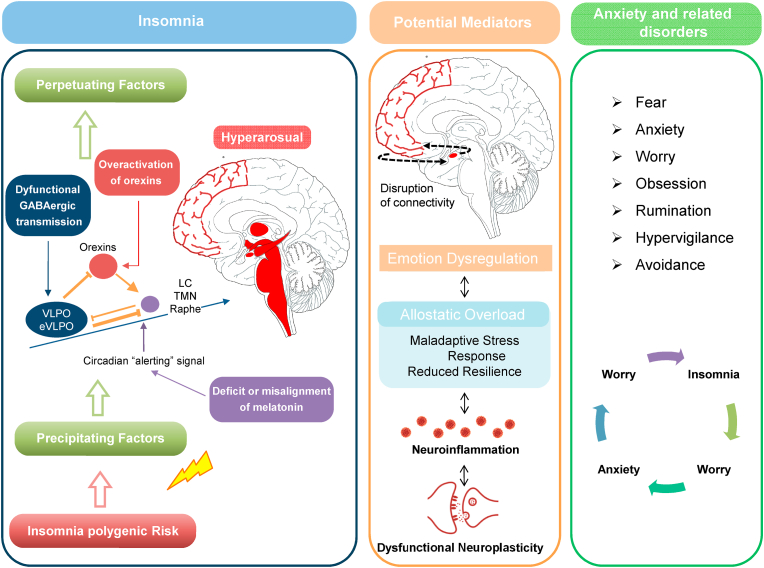
Potential Role of Insomnia in Anxiety and related disorders.

Through imaging studies, inflammation can also have an impact on connectivity in a variety of brain structures, and in particular on some regions associated with anxiety including anterior cingulate cortex, amygdala and insula (Felger, 2018). Stress response activation and cytokine release from central and peripheral immune cells may account for increased inflammation in PTSD, GAD, PD, and phobias. (Akkaoui et al., 2023) it is possible that insomnia may contribute to anxiety and related disorders favouring inflammation (Fig. 2).

Melatonin is an unusually multitasking molecule that participates in an uncountable number of physiological and pathological actions (Palagini et al., 2023b). Although the modulation of circadian rhythms is considered to be a principal role of melatonin, the melatoninergic anxiolytic effect have been confirmed in several experiments (for an overview, see Repova et al., 2022) (Repova et al., 2022). Since Melatonin alterations have been hypothesized in insomnia, (Palagini et al., 2023b) it is possible that insomnia may exert and anxiogenic effect through melatonin dysregulation.

### 4.4 Cognitive vulnerability

Cognitive theories of both anxiety and insomnia highlight the idea that dysfunctional behaviors develop or are maintained in part due to an individual's maladaptive cognition.

A cognitive model of insomnia proposed by Harvey (2002) (Harvey, 2002) proposed a reciprocal relationship between worrying in anxiety and insomnia. Specifically, insomnia is characterized by excessive worrying about sleep and the consequences of not sleeping leads to autonomic arousal and emotional distress and exacerbates insomnia. These cognitive an emotional aspect of insomnia may fuel worry, negative thinking, distress and dysfunctional behaviours in anxiety and related disorder. Insomnia-related worry, rumination, racing thoughts, and repetitive negative thinking may contribute to dysfunctional cognition of GAD, PD, PTSD, social phobia and OCD Insomnia-related dysfunctional beliefs may fuel the fear of loss of vigilance, which may be a cognitive component of PD, GAD and PTSD hence contributing to their perpetuation (Werner et al., 2021).

## 5 Conclusions

A previous systematic review was conducted on the potential association between sleep disturbances and anxiety/anxiety related disorder (Cox and Olatunji, 2016). Findings suggested that sleep disturbances exacerbate symptom severity in the majority of anxiety and related disorders. However, the nature of the association may vary as a function of different sleep disturbances (Cox and Olatunji, 2016). Hence, we decided to just focus on one sleep disturbances, such as insomnia. Our systematic search showed that although the majority of current data focus on GAD and PTSD, insomnia frequently co-occurs with a wide range of anxiety-related disorders, including PD, social phobia and OCD. It may negatively affect anxiety and related disorders trajectory linking anxiety to other mental disorders such as mood or substance use disorders and may increase suicidal risk. Potential mechanisms recognize the central role of insomnia-related hyperarousal and dysfunctional cognition, which may favor maladaptive stress-response, and emotion dysregulation and may fuel the cycle of worry in anxiety. Genetic, neuroanatomical and neuromodulating factors may overlap but a specific role of insomnia in anxiety and related disorders has recently recognized (Fig. 2). In particular, it is emerging the role of orexin over activation in both insomnia and anxiety with the potentiality of targeting anxiety and related disorders via sleep with DORAs, while gabaergic via may be not indicated in the context of anxiety and related disorders (Guina et al., 2015). Although more studies are needed on insomnia treatment in the context of anxiety and anxiety related disorders, targeting insomnia may be a key factor for improving anxiety and related disorder trajectories.

Future systematic review should focus on different sleep disturbances which may be comorbid in anxiety and related disorders including the role of circadian rhythms, parasomnia, Trauma Associated Sleep Disorder and sleep disorder breathing to clarify their potential role.

Insomnia is a complex polygenic stress-related disorder, and it is likely to be caused by a synergy of genetic and environmental factors within an epigenetic framework. Predisposing factors interact with precipitating factors for insomnia development and perpetuating factors contribute to chronic forms. The hyperarousal, that is the activation of arousal promoting neurotransmission consistent with the central and peripheral hyperactivation of stress and inflammatory systems, has been shown in insomnia. The amygdala-based intrinsic emotional network is abnormal, with decreased brain volume in the medial frontal and middle temporal gyrus, middle cingulate cortex, and hippocampus. Insomnia is therefore considered a stressor, which impairs neuroplasticity leading to a state of allostatic overload with an anxiogenic effect. These may result in disturbances with emotion regulation and decision-making processes, leading to emotional reactivity, impulsivity, aggressive behaviours, and risky decisions. The hyperactivity of the stress axis has consistently been demonstrated to be related to a functional reduction of the cortical GABAergic system and with deficient GABAergic synapses associated with cortical hyperarousal in insomnia. Maladaptive changes in neuroplasticity are hypothesized in insomnia, reflecting GABAergic alterations and dysfunctions in melatonin production. Data support the hypothesis that insomnia can be due to an inability of the brain to switch off wake-promoting systems such as the orexin system, as well as an inability to switch on sleep-promoting circuits with an instability of the flip-flop switch system^.^ Accordingly, it has been hypothesized that the overactivation of the orexin system in insomnia may favor the instability of the flip-flop switch system with the hyperactivation of arousal promoting systems, which may fuel hyperarousal. These factors may favor the cycle of worry and anxiety and fuel anxiety-related symptoms. VLPO = ventrolateral preoptic nucleus, LC = Locus Coeruleus, *TMN* = Tuberomammillary nucleus.

## Declaration of competing interest

None.
